# Drebrin Regulates Neuroblast Migration in the Postnatal Mammalian Brain

**DOI:** 10.1371/journal.pone.0126478

**Published:** 2015-05-06

**Authors:** Martina Sonego, Michelle Oberoi, Jake Stoddart, Sangeetha Gajendra, Rita Hendricusdottir, Fazal Oozeer, Daniel C. Worth, Carl Hobbs, Britta J. Eickholt, Phillip R. Gordon-Weeks, Patrick Doherty, Giovanna Lalli

**Affiliations:** 1 Wolfson Centre for Age-Related Diseases, King’s College London, London, United Kingdom; 2 Department of Anatomy and Neurobiology, University of California Irvine, Irvine, California, United States of America; 3 MRC Centre for Developmental Neurobiology, King’s College London, London, United Kingdom; 4 Cluster of Excellence NeuroCure and Institute of Biochemistry, Charité —Universitätsmedizin Berlin, Berlin, Germany; McGill University Department of Neurology and Neurosurgery, CANADA

## Abstract

After birth, stem cells in the subventricular zone (SVZ) generate neuroblasts that migrate along the rostral migratory stream (RMS) to become interneurons in the olfactory bulb (OB). This migration is crucial for the proper integration of newborn neurons in a pre-existing synaptic network and is believed to play a key role in infant human brain development. Many regulators of neuroblast migration have been identified; however, still very little is known about the intracellular molecular mechanisms controlling this process. Here, we have investigated the function of drebrin, an actin-binding protein highly expressed in the RMS of the postnatal mammalian brain. Neuroblast migration was monitored both in culture and in brain slices obtained from electroporated mice by time-lapse spinning disk confocal microscopy. Depletion of drebrin using distinct RNAi approaches in early postnatal mice affects neuroblast morphology and impairs neuroblast migration and orientation *in vitro* and *in vivo*. Overexpression of drebrin also impairs migration along the RMS and affects the distribution of neuroblasts at their final destination, the OB. Drebrin phosphorylation on Ser142 by Cyclin-dependent kinase 5 (Cdk5) has been recently shown to regulate F-actin-microtubule coupling in neuronal growth cones. We also investigated the functional significance of this phosphorylation in RMS neuroblasts using *in vivo* postnatal electroporation of phosphomimetic (S142D) or non-phosphorylatable (S142A) drebrin in the SVZ of mouse pups. Preventing or mimicking phosphorylation of S142 *in vivo* caused similar effects on neuroblast dynamics, leading to aberrant neuroblast branching. We conclude that drebrin is necessary for efficient migration of SVZ-derived neuroblasts and propose that regulated phosphorylation of drebrin on S142 maintains leading process stability for polarized migration along the RMS, thus ensuring proper neurogenesis.

## Introduction

The subventricular zone (SVZ), the largest neural stem cell niche of the postnatal mammalian brain, gives rise to new neurons throughout adulthood [1]. In the rodent brain, SVZ-derived neural progenitors migrate tangentially in chains along the rostral migratory stream (RMS) towards the olfactory bulb (OB). Once in the OB, the chains disperse and neuroblasts migrate radially to differentiate into interneurons able to integrate into the pre-existing synaptic circuit [2–4]. The SVZ is also a major neurogenic niche in the postnatal human brain [5]. Neuroblast migration along the RMS is prominent in human infancy, and is believed to play an important role at this crucial stage of brain development [6]. In adulthood, human SVZ-derived neuroblasts migrate towards the striatum, and this process is impaired in Huntington’s disease patients [5]. Migratory neuroblasts also have the ability to target injured areas [7]. While a plethora of extracellular factors, extracellular matrix components and neurotransmitters can regulate SVZ-derived neuroblast migration [8], the intracellular molecular mechanisms underlying this important process in neurogenesis remain obscure.

In this work we investigate the role of drebrin, an actin-binding protein, in SVZ-derived neuroblast migration. Two drebrin isoforms (drebrin E and drebrin A) are produced from a single gene by alternative splicing and differ by an additional internal sequence present in drebrin A [9]. While drebrin E is ubiquitously expressed and is highly abundant in the developing brain, drebrin A is neuron-specific and predominates in the adult forebrain [10–12]. In developing cortical neurons drebrin E regulates neuritogenesis by coordinating F-actin-microtubule interactions taking place in growth cones, due to its ability to bind F-actin-rich filopodia and the plus-tip microtubule-associated protein EB3 [13]. Interestingly, drebrin’s role in neurite outgrowth is regulated by Cdk5, a pivotal kinase involved in neuronal migration in the developing brain and also in RMS neuroblast motility [14]. Indeed, Cdk5-dependent phosphorylation of drebrin on S142 activates drebrin’s actin-bundling function and facilitates microtubule binding, enhancing neuritogenesis [15]. A recent report has shown that drebrin is necessary for the migration of oculomotor neurons, where it is involved in the formation and correct orientation of the leading process [16]. In glioma cells, drebrin localizes at the leading edge of lamellipodia and regulates cell morphology as well as cell motility [17]. The reversible phosphorylation and dephosphorylation of drebrin by Cdk5 on Ser142 is also important for the radial glia-guided migration of neurons in the developing mammalian cortex [18]. Interestingly, drebrin E is highly expressed in the RMS, but is downregulated once newborn neurons stop migrating to differentiate in the OB [19], suggesting an important function for this protein in controlling the motility of SVZ-derived neuroblasts. However, the role of drebrin in neuroblast migration is still completely unknown.

Here, we show that drebrin is highly expressed in postnatal SVZ-derived migratory neuroblasts. Using RNAi and overexpression approaches (including nucleofection of primary neuroblasts and *in vivo* postnatal electroporation) we show that altering drebrin levels affects tangential migration along the RMS and neuroblast distribution in the OB. An important role for phosphorylation of drebrin on serine 142 (S142) is indicated by the observation that expression of a non-phosphorylatable drebrin mutant (S142A), or a phosphomimetic drebrin mutant (S142D), causes misorientation defects and similar aberrant branching of the leading process of migratory neuroblasts, as shown by time-lapse imaging of brain slice cultures from mice electroporated with drebrin phospho-mutants. We conclude that tightly regulated levels of drebrin are necessary for proper migration of SVZ-derived neuroblasts to ensure efficient neurogenesis in the postnatal mammalian brain.

## Materials and Methods

### Antibodies and reagents

The following antibodies were used: mouse/rabbit anti-drebrin, mouse/rabbit anti-βIII Tubulin, rabbit anti-doublecortin (DCX) (Abcam); rabbit anti-actin (Cell Signaling), anti-GFAP (Dako), anti-GFP (Invitrogen), rabbit anti-pS142 drebrin [15]; mouse anti-PSA-NCAM (Sigma). HRP-conjugated antibodies were from Thermo Scientific. Biotinylated HRP antibodies were from Dako. Alexa Fluor 488- and Texas Red-conjugated secondary antibodies were from Invitrogen. Unless otherwise specified, all chemicals were from Sigma and all cell culture reagents were from Invitrogen.

### siRNA and plasmids

The following DNA plasmids were used: pCX-EGFP was a kind gift from Dr. Masaru Okabe and Jun-ichi Miyazaki (Osaka University, Japan), drebrin pCAG-shRNA-IRES-GFP, sequence AGAACCAGAAAGTGATGTA [20], human drebrin E-mCherry-N1, pCAG empty vector-YFP, pCAG-wild type human drebrin E-YFP, pCAG-drebrinS142A-YFP, and pCAG-drebrinS142D-YFP [15].

Smart pools of four pre-designed small interfering RNA (siRNA) oligos targeting rat drebrin and control siRNA oligos (Dharmacon) were dissolved in 1X siRNA buffer (containing: 60 mM KCl, 6 mM HEPES-pH 7.5, and 0.2 mM MgCl_2_) to a final concentration of 20 μM. The oligo sequences of the smartpool targeting rat drebrin were as follows: 5’GGUGAUUAGUAGUGGCGAC3’, 5’GGUUUGAGCAGGAGCGGAU3’, 5’CCUGAUAACCCACGGGAGU3’, and 5’CUGAAUUCUUCCAGGGCGU3’.

### Animals

All the procedures were approved by the Committee on the Ethics of Animal Experiments of King’s College London and performed in accordance with UK Home Office Regulations (Animal Scientific Procedures Act, 1986). P2-P3 CD1 mouse pups (Charles River) and P6-P7 Sprague Dawley rat pups (Harlan) of both sexes were used. Sagittal brain sections for immunohistochemical analysis were obtained from P7 and P90 CD-1 mice. Animals were sacrificed by cervical dislocation. *In vivo* postnatal electroporation of P2-P3 CD1 mouse pups was performed under isofluorane anesthesia and all efforts were made to minimize suffering.

### *In vitro* neuroblast migration assay

#### Nucleofection, reaggregation and embedding

Dissociated neuroblasts from rat RMS tissue were pelleted and re-suspended in rat neuron nucleofection solution (Lonza) at a final concentration of 3x10^6^ cells/100 μl. Each sample was mixed with either 5/9 μg of siRNA or 3/5 μg of shRNA plasmid, and nucleofected using program G-013. The cell suspension was then supplemented with 6 ml of DMEM + 10% FCS, and spun at 1,500 rpm for 5 minutes. The resulting pellet was resuspended in 25 μl of DMEM + 10% FCS, and pipetted as a hanging drop over a 35 mm dish containing 2 ml of complete culture medium pre-equilibrated at 37°C/5% CO_2_. After 5 hours at 37°C/5% CO_2_, the cell reaggregate was transferred into the medium, and cultured in suspension at 37°C/5% CO_2_ for either 24 or 48 hours. Cell reaggregates were then embedded in growth factor-reduced, phenol red-free Matrigel (Becton Dickinson) diluted 3:1 in Neurobasal complete medium as previously described [21]. Explants were left to migrate for up to 24 hours at 37°C/5% CO_2_. In some experiments BTP (1 μM or 2 μM) was added to the Matrigel as well as to the culture medium.

#### Immunocytochemistry

Neuroblast reaggregates were fixed at room temperature with 4% PFA in PBS, washed three times with PBS, and blocked with 5% goat serum, 0.3% Triton X-100, 0.1% BSA in PBS for 1 hour at room temperature. Coverslips were washed and incubated with primary antibodies diluted in blocking solution overnight at 4°C. After washing, coverslips were incubated with secondary antibodies diluted in blocking solution with Hoechst for 2 hours at room temperature, washed and mounted with fluorescent mounting medium (Dako). Pictures of explants were taken on a Zeiss Axioplan 2 microscope equipped with an ApoTome module and a Zeiss MRm Axiocam CCD camera using A-Plan 10x/0.25 and Plan Apo 20x/0.75 objectives and Axiovision software. The migration distance was measured from the edge of the explants to the nucleus of the furthest migrated cell (identified by Hoechst staining) for at least 6 different positions around the explants using ImageJ as described [21]. Data were collected from 3 independent experiments, analysing at least 15 explants per condition in each experiment.

### *In vivo* postnatal electroporation

P2-P3 CD1 mouse pups were anesthetized with isofluorane for 1 minute. Using a pulled glass capillary, 3 μl of a 1 μg/μl plasmid stock were injected into the right lateral ventricle. Animals were then subjected to five electrical pulses of 99.9 V for 50-milliseconds with 850-milliseconds intervals using the CUY21SC electroporator (Nepagene) and 7 mm tweezer electrodes coated with conductive gel (CEFAR, France) [22]. Pups were then reanimated under oxygen and returned to their mother. Brains were collected 5 days later for morphological analysis of neuroblasts along the RMS or 14 days later for analysis of neuroblast distribution in the OB.

### Immunohistochemistry

#### Gelatin-embedded sections

Brains were fixed in PBS containing 4% PFA at room temperature for 3 hours, embedded in gelatin as previously described [23] and cut into 50 μm-thick sagittal slices for immunostaining. Slices were blocked for 1 hour in PBS containing 1% BSA, 0.1% Triton X-100, incubated with primary antibodies overnight at 4°C on roller, washed in PBS and incubated with fluorescent secondary antibodies and Hoechst (1:5000) for 2 hours at room temperature. After washing, slices were mounted in fluorescent mounting medium.

#### Paraffin-embedded sections

Formalin-fixed P7 or adult mouse brains were embedded in paraffin and cut into 6 μm-thick sagittal sections. Brain sections were deparaffinised and rehydrated before heat-induced antigen retrieval using a sodium citrate buffer. Slices were blocked in Tris-buffered saline (TBS) 50 mM pH 7.6 containing 1% BSA and 0.1% sodium azide and incubated with primary antibodies diluted in the same solution at 4°C overnight. Further processing with biotinylated secondary antibodies and counterstaining with hematoxylin was performed as previously described [23].

For immunofluorescence sections were deparaffinised, blocked with 1% BSA for 15 min, and incubated with primary antibodies overnight at 4°C. Sections were then incubated with appropriate fluorescent secondary antibodies and Hoechst dye for 1 hour at room temperature and mounted as described [23].

### Analysis of neuroblast morphology in the RMS

The morphology of GFP/YFP-expressing neuroblasts was examined in confocal z-stack projections of 50 μm-thick sagittal brain slices immunostained with an anti-GFP/YFP antibody 5 days after electroporation. Z-stacks (images taken every 0.5–1.5 μm) of the RMS were captured on a Zeiss LSM 710 confocal microscope using an EC Plan Neofluar 40x/1.3 objective and Zeiss Zen software. Process length was measured as the distance from the base of the cell body to the tip of the leading process using ImageJ. Cells were considered with secondary branches if they displayed one or more protrusions extending from the leading process and from the cell body. To analyze orientation, we traced a line in the middle of the cell body running perpendicular to the direction of migration along the RMS towards the OB. Neuroblasts with their leading process extending in the sector opposite to the direction of migration were considered misoriented. At least 250 cells were analyzed from each brain, and between 3 and 6 brains were analyzed for each electroporated construct/genotype.

### Analysis of neuroblast distribution in OB

To analyze the distribution of YFP-labeled cells in the OB, a mask outlining the inner (“A”) and outer (“B”) OB areas (minor axis: 1.25 and 3 mm; major axis: 0.73 and 1.7 mm, respectively) was aligned with the edge of the OB on confocal projections of 50 μm-thick sagittal brain slices [24]. For each brain, the percentage of cells in the two OB areas was calculated after counting GFP-positive cells in all the slices containing the OB (typically 4–5 slices/brain). Three brains were analyzed for each electroporated plasmid.

### Brain slice culture

Five days after electroporation, electroporated brain hemispheres were sagitally sectioned into 300 μm-thick slices with a Leica VT1000S Vibratome. Slices containing fluorescently-labelled neuroblasts along the full RMS were chosen and cultured for 1 hour on a Millicell insert (Millipore) placed in a p35 glass-bottom dish (MatTek) containing brain slice imaging medium (phenol red-free DMEM) supplemented with 5% FCS, 0.5% glucose, 4 mM glutamine, B27 glutamine, B27 supplement, 10 mM HEPES (pH 7.4), 100 units/ml penicillin and 100 μg/ml streptomycin) [22].

### Tracking time-lapse imaging of brain slices

Cultured brain slices were transferred into a pre-heated (37°C) chamber of a Perkin Elmer UltraView VoX confocal spinning disk system. Time-lapse imaging of GFP positive cells in the RMS was performed using an inverted Nikon Ti-E microscope with a Nikon CFI Super Plan Fluor ELWD 20x/0.45 objective coupled with a Hamamatsu C10600-10B (ORCA-R2) cooled digital CCD camera every 3 minutes for a total period of 3 hours. Z-stack images were taken every 4 μm over an interval of 100–150 μm inside the brain slice [22]. Movies were acquired and analyzed using Volocity software (Perkin Elmer). The dynamics of fluorescently-labelled neuroblasts was quantitatively analyzed by tracking the cell body of each neuroblast present in the field of view throughout the entire duration of imaging. The following parameters were analyzed: migrated distance (μm), velocity (μm/h), displacement (μm), and migratory index (ratio between the net displacement and the total distance travelled) [25]. Only cells that had a displacement of at least 50 μm were considered. Between 15 and 30 neuroblasts were tracked in each movie, and slices from 5 different brains per condition were analyzed.

Branching events per hour were quantified by frame-by-frame visual analysis of time-lapse movies. For each condition, between 17 and 32 cells per brain were analyzed from a total of 3–6 brains per electroporated plasmid.

### Western Blotting

Nucleofected neuroblasts were plated in 35 mm plates coated with 0.5 mg/ml polyornithine and 10 μg/ml laminin. After 2 or 3 days, cells lysates were prepared, run on a 8% SDS-polyacrylamide gel and analysed by Western blotting as previously described [23].

### Statistical Analysis

Statistical analysis was performed using Student’s *t*-test for dual comparison and one-way ANOVA for multiple comparisons with SigmaPlot 12.0 (Systat Software Inc). Differences were considered statistically significant if p < 0.05. In all figures: *P<0.05, **P <0.01, ***P<0.001. Error bars in all the graphs represent the standard error of the mean (SEM). Two-tailed comparisons were used in all the experiments.

## Results

### Drebrin is highly expressed in postnatal SVZ-derived migratory neuroblasts

**Fig 1:**
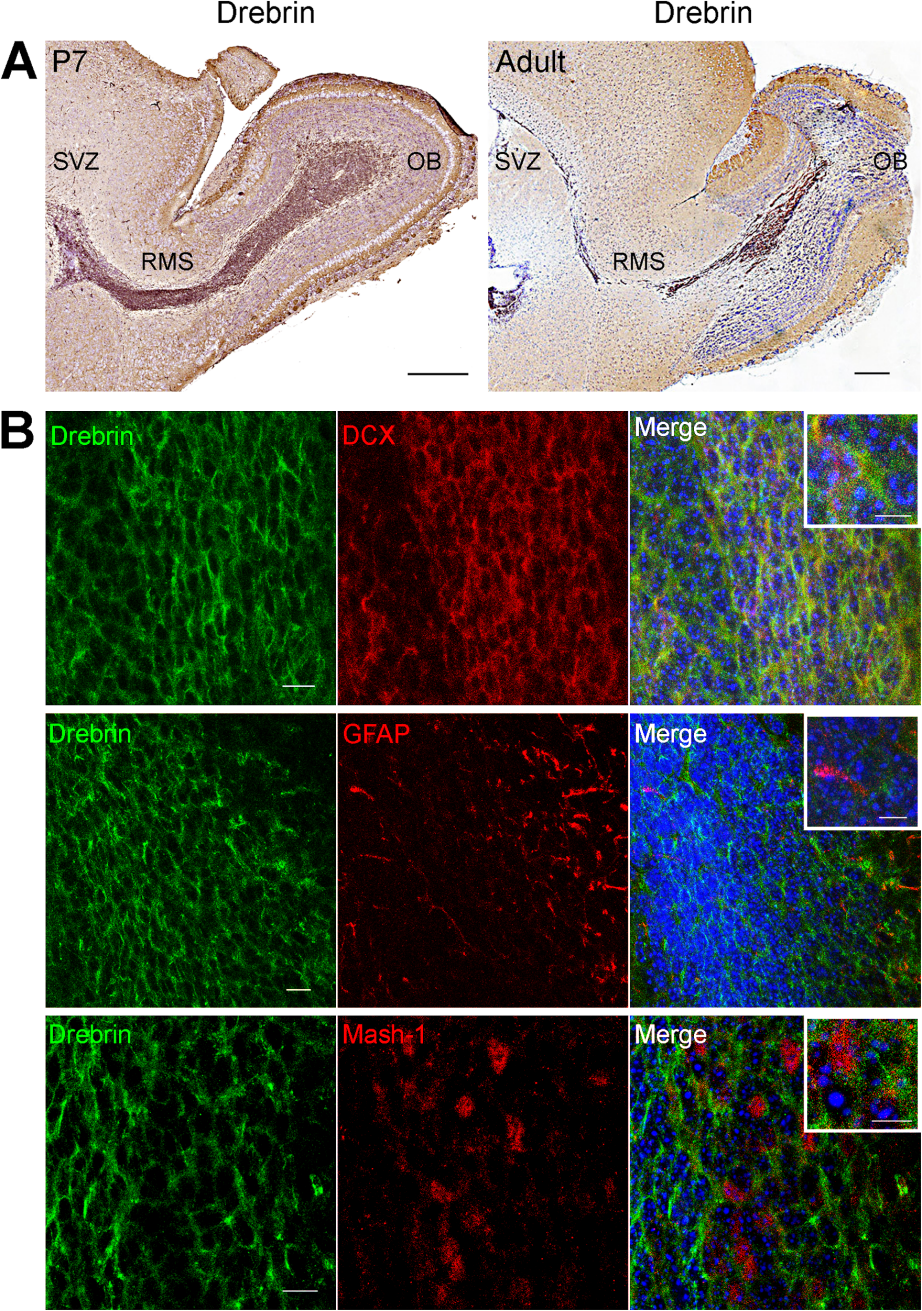
Drebrin is highly expressed in SVZ-derived migratory neuroblasts. (A) Immunostained sagittal brain sections from P7 (left) and P90 (right) mice show strong drebrin expression (brown) in the subventricular zone (SVZ), rostral migratory stream (RMS), and olfactory bulb (OB). (B) Confocal images from P7 mice SVZ sagittal sections showing that drebrin immunostaining overlaps with DCX+ migrating neuroblasts (top row), but is almost completely excluded from GFAP+ stem cells and astrocytes (middle row). Very little colocalization is observed with Mash-1+ transit-amplifying progenitors (bottom row). Merge panels include DAPI staining to visualize cell nuclei and higher magnification insets. Scale bars: (A), 200 μm; (B), 10 μm; insets, 5 μm.

We analysed the distribution of drebrin in paraffin-embedded P7 and P90 mouse sagittal brain slices (Fig 1A). At both time points drebrin is highly expressed in the RMS, resembling the expression pattern of PSA-NCAM and DCX, two well-characterised RMS neuroblast markers [26]. A previous study reported that drebrin is present in the adult rat SVZ-RMS [19] and that drebrin-positive cells also express the migrating neuroblast marker PSA-NCAM, with some co-localization with the proliferating cell marker Ki67 [27, 28]. However, no co-expression was detected with the astrocytic stem cell marker GFAP [19]. Consistent with these observations, double immunostaining of coronal mouse SVZ sections showed almost complete co-localization of drebrin with the migrating neuroblast marker DCX (Fig 1B, top row), while very little co-localization was observed with GFAP (Fig 1B, middle row) and with the transit amplifying progenitor marker Mash-1 (Fig 1B, bottom row).

**Fig 2:**
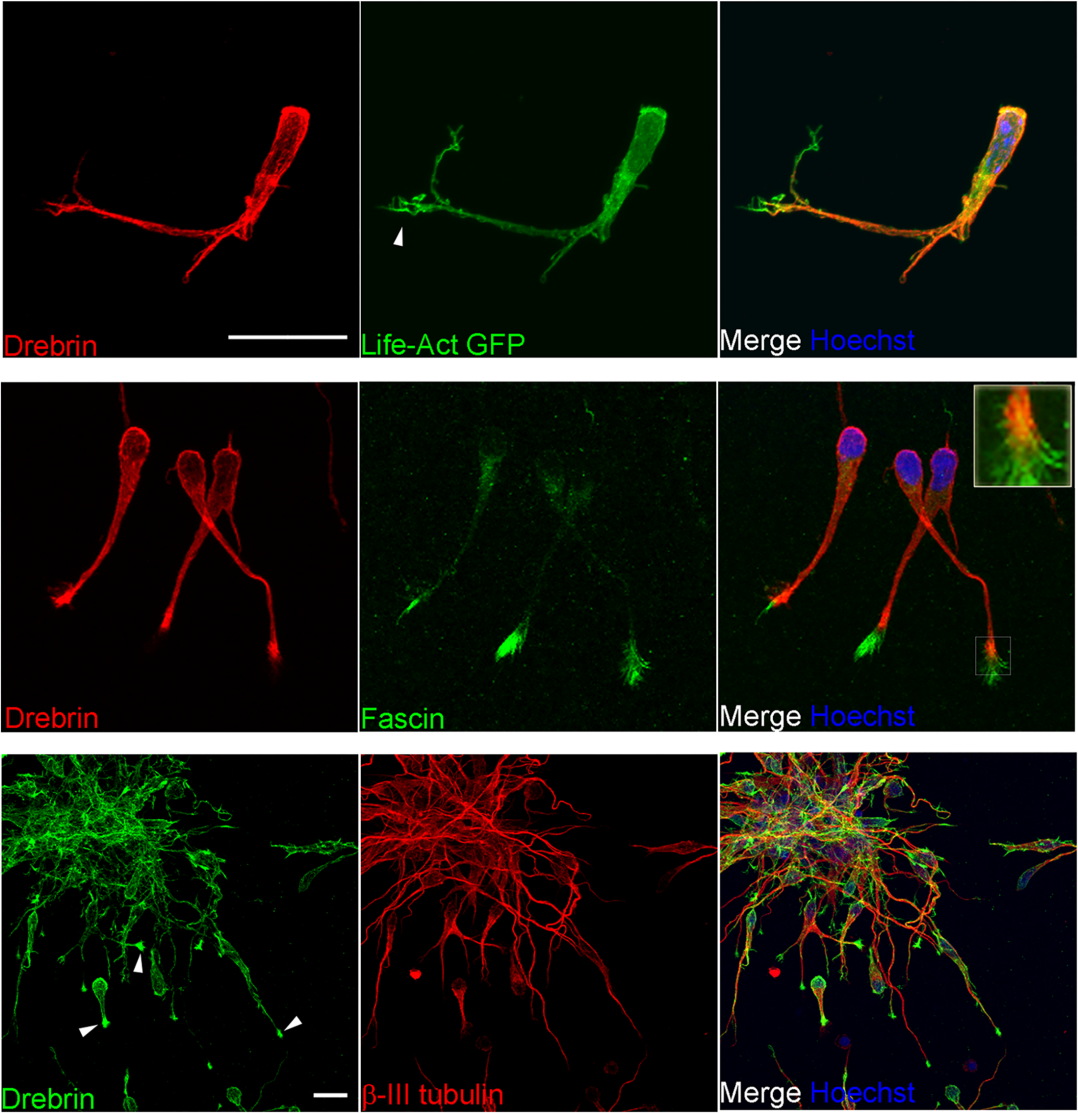
Intracellular localisation of drebrin in migratory neuroblasts. Immunostaining of cultured rat RMS neuroblasts embedded in Matrigel. (Top row) Drebrin (red) is present along the leading process but mostly excluded from peripheral filopodia, visualized by Life-Act GFP (green, arrowhead). Nuclei are stained with Hoechst dye (blue). (Middle row) Colocalization between drebrin (red) and fascin (green), which decorates peripheral filopodia, is limited to the basal region of filopodia. (Bottom row) Drebrin (green) is found along the membrane and especially concentrated towards the tip of leading processes (arrowheads), which are immunostained for ßIII tubulin-positive microtubules (red). Nuclei are stained with Hoechst dye (blue). Scale bars: (top), 10 μm; (middle and bottom), 20 μm.

We examined the intracellular distribution of drebrin by immunostaining neuroblasts in RMS explants embedded in a three-dimensional Matrigel matrix, a substrate allowing neuroblast migration [29]. Drebrin was detected along the leading process, and was especially concentrated in an area of the process tip partially overlapping with the basal region of terminal filopodia, which were visualized by transfection of Life-Act GFP [30] (Fig 2, top row, arrowhead). Interestingly, the basal filopodia region also displayed some colocalisation between drebrin and fascin, an actin-bundling protein present in terminal filopodia and required for efficient neuroblast migration [23]. However, drebrin seemed to be excluded from the peripheral portion of these filopodia (Fig 2, middle row and inset). In addition, drebrin appeared to be located at the distal tip of ßIII tubulin-positive microtubules in the neuroblast leading process (Fig 2, bottom row, arrowheads). Therefore, drebrin is highly expressed in migratory neuroblasts and especially in a region of the leading process tip encompassing microtubule ends and the basal portions of terminal filopodia, an area of active cytoskeletal rearrangement.

### Drebrin knockdown affects neuroblast morphology *in vitro* and *in vivo*

**Fig 3:**
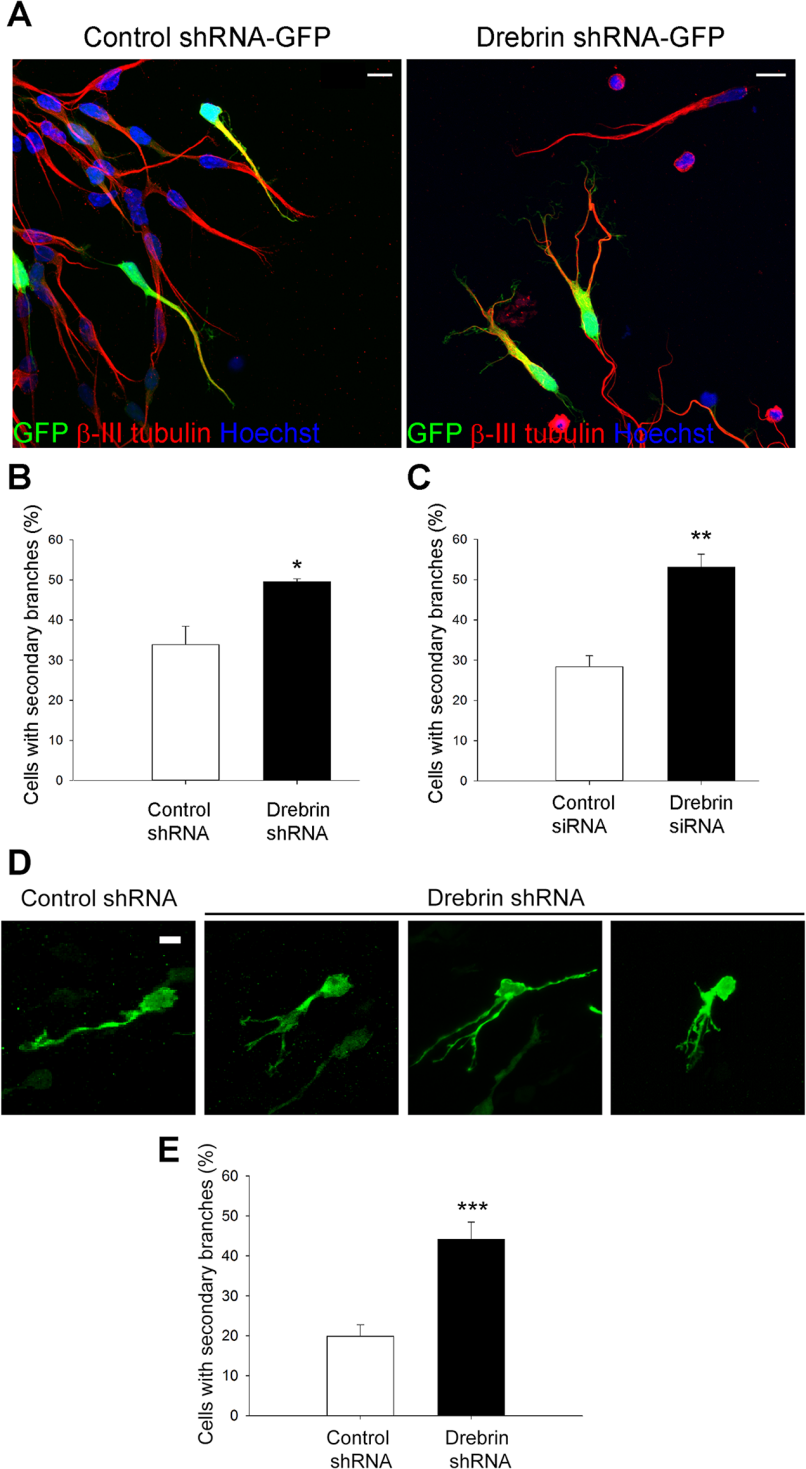
Drebrin knockdown affects neuroblast morphology *in vitro*. (A) Representative images of rat RMS neuroblasts nucleofected with control (left) or drebrin (right) shRNA-GFP. Note the branched morphology in drebrin shRNA-transfected cells (right, yellow), compared to the unipolar shape of control neuroblasts (left, yellow). (B) shRNA- and (C) siRNA-mediated drebrin depletion significantly increases the percentage of branched cells (mean ± SEM; n = 3 independent experiments; 191 cells analysed for control shRNA, 192 cells for drebrin shRNA; 202 cells for control siRNA, 207 cells for drebrin siRNA; *P<0.05; **P<0.01). (D) P2 mouse pups were electroporated in the lateral ventricle with control or drebrin shRNA-GFP plasmids. Sagittal brain slices containing the RMS were immunostained for GFP 5 days later. Confocal projections showing a typical control cell extending a single process oriented towards the OB, and representative drebrin shRNA-transfected neuroblasts displaying branched protrusions. (E) *In vivo* postnatal electroporation of drebrin shRNA causes a significant increase in the percentage of branched neuroblasts (mean ± SEM; n = 6 brains per condition, 400 cells analysed for control shRNA and 303 cells analysed for drebrin shRNA; ***P<0.001). Scale bars: (A), 10 μm; (D), 5 μm.

To characterize the role of drebrin in neuroblasts, we first examined neuroblast morphology in drebrin-depleted cells. RMS neuroblasts were nucleofected with drebrin shRNA-GFP, an approach we have successfully used to knock down proteins of interest in neuroblasts [21, 23, 24], or with control shRNA. Cells were then cultured in suspension for 52 h, embedded in Matrigel and subsequently left to migrate for a period of 24 h, before immunostaining with anti-GFP and -βIII tubulin antibodies. We achieved considerable drebrin depletion (~80%) at this time point, as monitored by Western blot of neuroblast lysates and immunostaining (S1 Fig). We were also able to achieve similar drebrin knockdown by siRNA oligo nucleofection (S1 Fig). Drebrin knockdown visibly affected neuroblast morphology (Fig 3A). While most control cells displayed a single straight leading process (Fig 3A, left), many drebrin-depleted cells had leading processes with multiple branches (Fig 3A, right). Indeed, drebrin depletion caused a ~30% increase in the percentage of neuroblasts with a branched morphology compared to control cells (Fig 3B). This effect was confirmed using nucleofection of siRNA oligos targeting drebrin as an alternative RNAi approach (Fig 3C), suggesting an important role for drebrin in regulating neuroblast morphology *in vitro*.

To test this role for drebrin *in vivo*, we electroporated control or drebrin shRNA-GFP plasmids in the lateral ventricle of P2 mouse pups, a technique allowing transfection of a neuroblast subpopulation in the intact SVZ [22, 31]. Brain slices were immunostained for GFP 5 days later to examine the morphology of SVZ-derived neuroblasts in the RMS by confocal microscopy (Fig 3D). To determine if drebrin was knocked down *in vivo*, neuroblast cultures were obtained by dissociating the RMS of the electroporated mouse right brain hemispheres. GFP-labelled cells had lower levels of drebrin immunostaining compared to control shRNA-transfected neuroblasts (data not shown). Electroporation of drebrin shRNA more than doubled the number of neuroblasts displaying secondary branches within the RMS (Fig 3E). Taken together, these data indicate that drebrin regulates the morphology of SVZ-derived neuroblasts.

### Drebrin is required for efficient neuroblast migration *in vitro* and *ex vivo*

It has been previously reported that defects in migration can be accompanied by a higher percentage of secondary branches [23, 29, 32]. We asked whether the high expression of drebrin in neuroblasts dictates a functional role for this protein in these highly migratory cells. To initially assess if drebrin is involved in neuroblast migration, we treated RMS explants with 3,5-bis(trifluoromethyl)pyrazole (BTP), an immuno-suppressant drug which binds to drebrin and inhibits F-actin binding [33]. BTP blocks store-operated calcium entry as well as actin rearrangements induced by drebrin [33, 34].

To test whether pharmacological inhibition of drebrin had an effect on neuroblast migration, P7 rat RMS explants were embedded in Matrigel and left to migrate for 18 hours in the presence of vehicle or BTP (1 μM). Incubation with BTP substantially impaired migration of neuroblasts out of RMS explants compared to control cells (S2 Fig). These results suggest that drebrin may have a role in neuroblast migration *in vitro*. We subsequently examined the effect of BTP-induced drebrin inhibition on neuroblast migration *ex vivo*. P2 mice were electroporated in the lateral ventricle with pCX-EGFP to label a subpopulation of RMS neuroblasts with GFP [29]. Five days later, neuroblast migration was monitored by time-lapse spinning disk confocal microscopy of acute brain slices cultured in medium with either control vehicle or BTP (1 μM or 2 μM). At both concentrations of BTP, neuroblasts migrating in the brain slice show a significant decrease in migrated distance, displacement, and velocity (S2 Fig). Interestingly, at the higher BTP concentration, the neuroblast average migratory index (i.e. the ratio between net displacement and total distance covered) significantly decreased compared to control cells (0.523 ± 0.027, n = 7 BTP-treated brain slices; 0.622 ± 0.030, n = 5 control brain slices; *P<0.05). This decrease was reflected in the fact that BTP-treated brain slices had a significantly lower percentage of “migratory” neuroblasts (i.e. displaying an efficient directed movement) compared to control samples (S2 Fig). These data suggest that pharmacological inhibition of drebrin using BTP impairs RMS neuroblast migration *ex vivo*.

**Fig 4:**
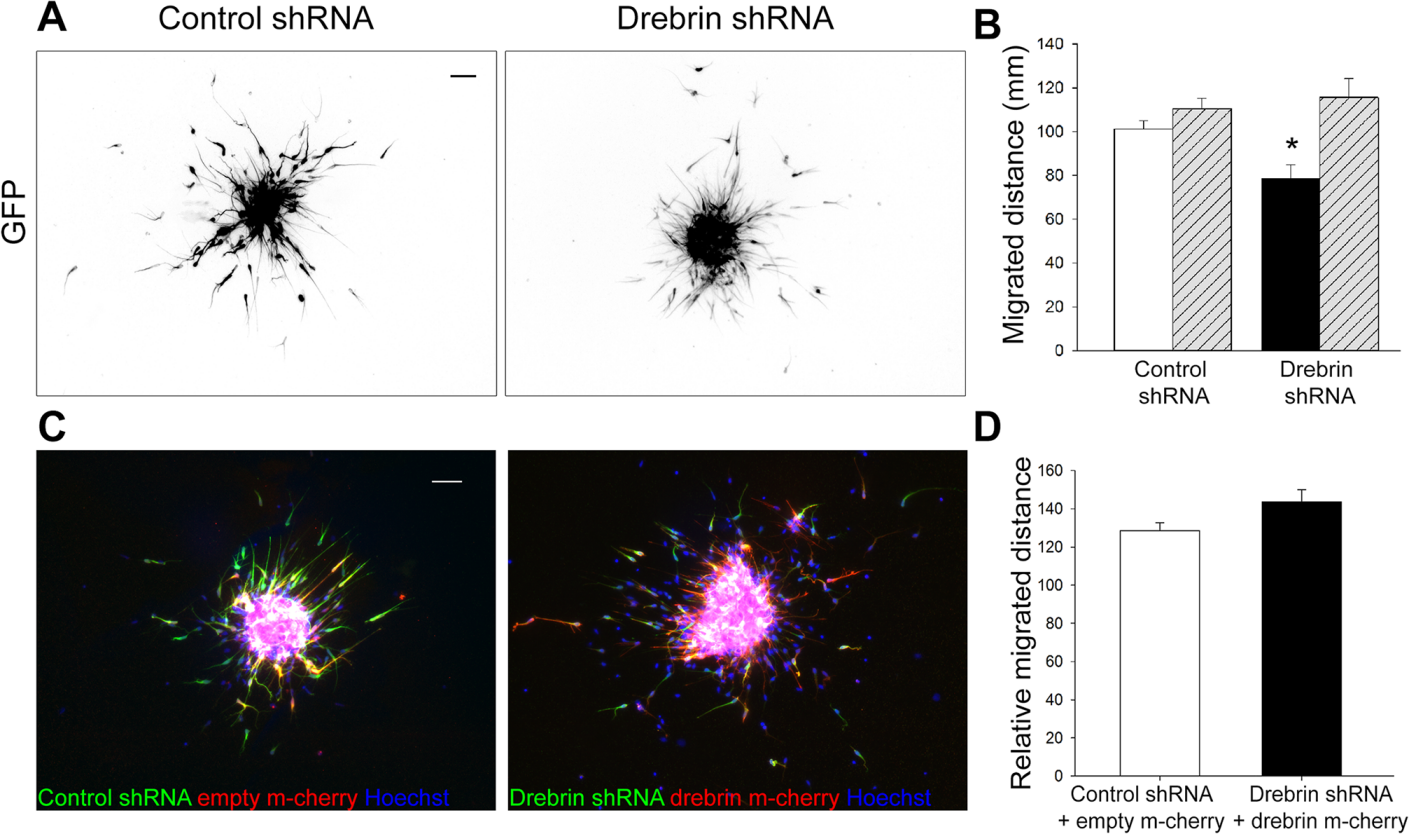
Drebrin regulates RMS neuroblast migration *in vitro*. (A) Reaggregated rat neuroblasts were embedded in Matrigel 52 h after nucleofection of control or drebrin shRNA-GFP and allowed to migrate for 24 h. Representative images of fixed reaggregates immunostained for GFP. The GFP channel is shown as a grayscale image. (B) Drebrin-depleted neuroblasts (black column) show a ~20% decrease in migration distance compared to control shRNA-nucleofected cells (white column). GFP-negative, untransfected cells (hatched columns) served as an internal control (mean ± SEM; n = 3 independent experiments; 15 to 20 explants analysed per experiment; *P<0.05). (C) Reaggregated rat neuroblasts were embedded in Matrigel 52 h after nucleofection with control shRNA-GFP and m-cherry-empty vector or drebrin shRNA-GFP and m-cherry-human drebrin (shRNA-resistant) and allowed to migrate for 24 h before immunostaining for GFP (green) and m-cherry (red). Cell nuclei were visualized by Hoechst staining (blue). (D) The impaired migration caused by drebrin knockdown was rescued by co-transfection with the shRNA-resistant human drebrin (mean ± SEM; n = 3 independent experiments; 15 to 20 explants analysed per experiment). Scale bars: 50 μm.

To gather further evidence for a role of drebrin in neuroblast migration, we used an *in vitro* migration assay [21]. Rat neuroblasts nucleofected with either control or drebrin shRNA-GFP were reaggregated in hanging drops and cultured in suspension for 52 h, subsequently embedded in Matrigel and left to migrate for 24 h before immunostaining for GFP (Fig 4A). As described above, we achieved efficient (80%) drebrin knockdown in the time allowed for neuroblast migration in this assay using this approach (S1 Fig). Quantitative analysis showed a ~25% decrease in migration distance for the drebrin-depleted cells compared to neuroblasts nucleofected with control shRNA (Fig 4B). Similar results were observed when cells were nucleofected with drebrin-targeting siRNA oligos (not shown). To prove that defective migration was specifically caused by the lack of drebrin, neuroblasts were nucleofected with drebrin shRNA together with a shRNA-resistant mCherry-tagged human drebrin (Fig 4C). Impaired migration by drebrin shRNA was prevented by co-transfecting human drebrin, confirming the specificity of the shRNA effect (Fig 4D). Taken together, these results show that drebrin is required for neuroblast migration *in vitro*.

**Fig 5:**
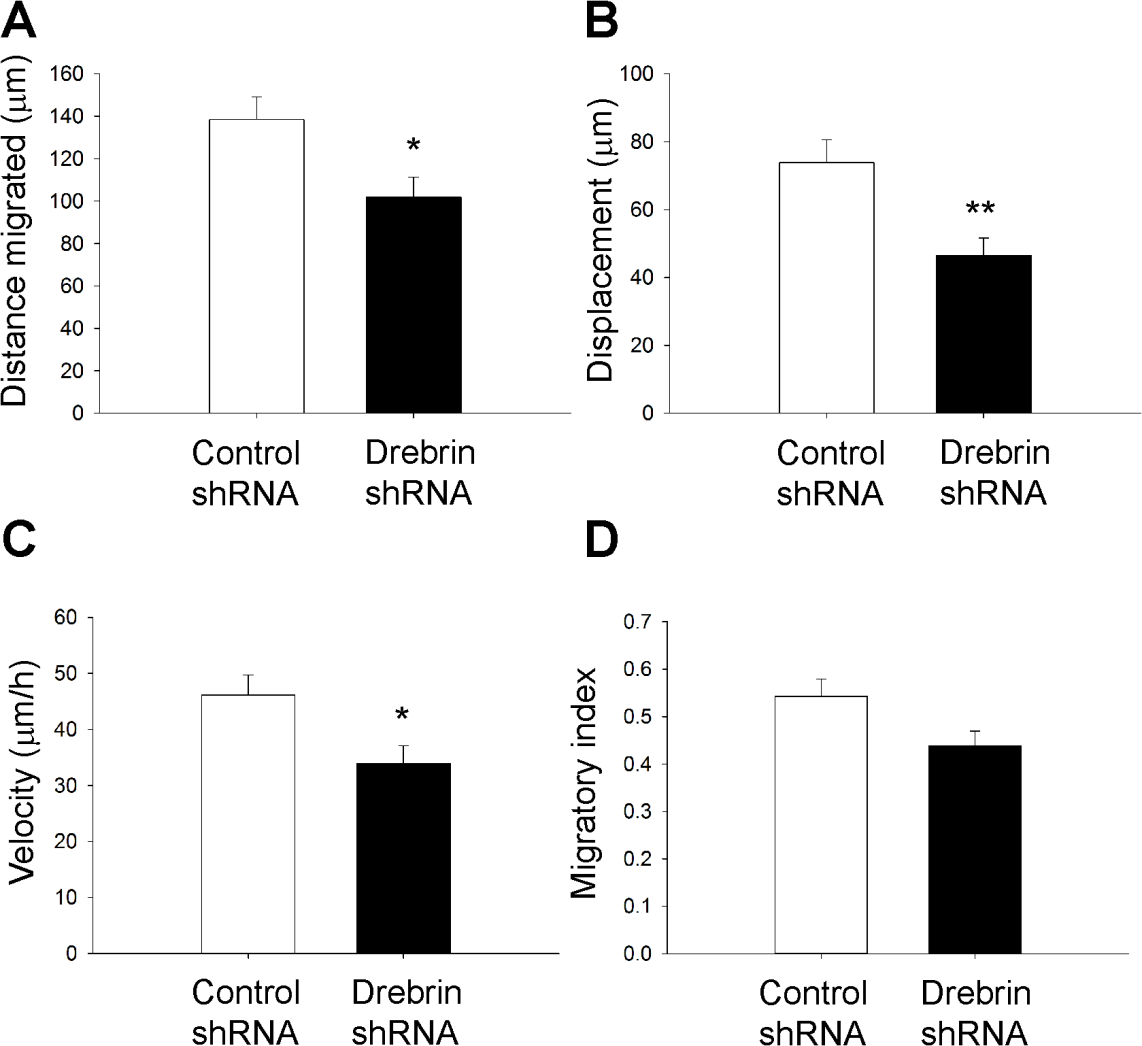
Drebrin is necessary for efficient neuroblast migration *ex vivo*. P2 mouse pups were electroporated in the lateral ventricle with control or drebrin shRNA-GFP. After 5 days, acute brain slice cultures were prepared and GFP-labelled neuroblasts were imaged by spinning disk confocal microscopy for 3 hours. (A-D) Tracking analysis shows that drebrin-depleted neuroblasts migrated over a shorter distance (A), with decreased overall displacement (B), speed (C) and a trend towards a decreased migratory index (D) (mean ± SEM; n = 8 slices for control; n = 7 slices for drebrin shRNA; *P<0.05; **P<0.01).

After determining an important role for drebrin in RMS neuroblast migration *in vitro* using two different RNAi approaches, we examined the effect of drebrin depletion on neuroblast migration within brain slices containing the RMS. We electroporated control or drebrin shRNA-GFP plasmids in the lateral ventricle of P2 mouse pups and visualized neuroblast dynamics by spinning disk confocal time-lapse imaging of acute brain slices cultures 5 days after electroporation. Compared to control shRNA cells, drebrin shRNA-transfected neuroblasts displayed reduced migrated distance (Fig 5A), displacement, (Fig 5B), and velocity (Fig 5C), while no significant difference was found in the migratory index (ratio between net displacement and total distance traveled) [35] (Fig 5D). This suggests that drebrin plays a cell-autonomous role in controlling neuroblast migration within the RMS.

### Drebrin phosphorylation on S142 regulates neuroblast orientation *in vivo*

After establishing that drebrin expression is necessary for efficient RMS neuroblast migration, we started to explore the molecular mechanisms by which drebrin controls neuroblast migration. We decided to examine a potential role for drebrin phosphorylation on Ser142, a crucial event coordinating the actin bundling function and microtubule-binding ability of this protein [15]. In developing neurons, phosphorylation of this site enables drebrin to bundle actin filaments and bind to microtubules via an interaction with the plus tip protein EB3, ultimately stimulating neuritogenesis [15]. This F-actin/microtubule cross-bridging activity of drebrin regulated by S142 phosphorylation could also play a role in regulating leading process dynamics during the migration of neuroblasts.

**Fig 6:**
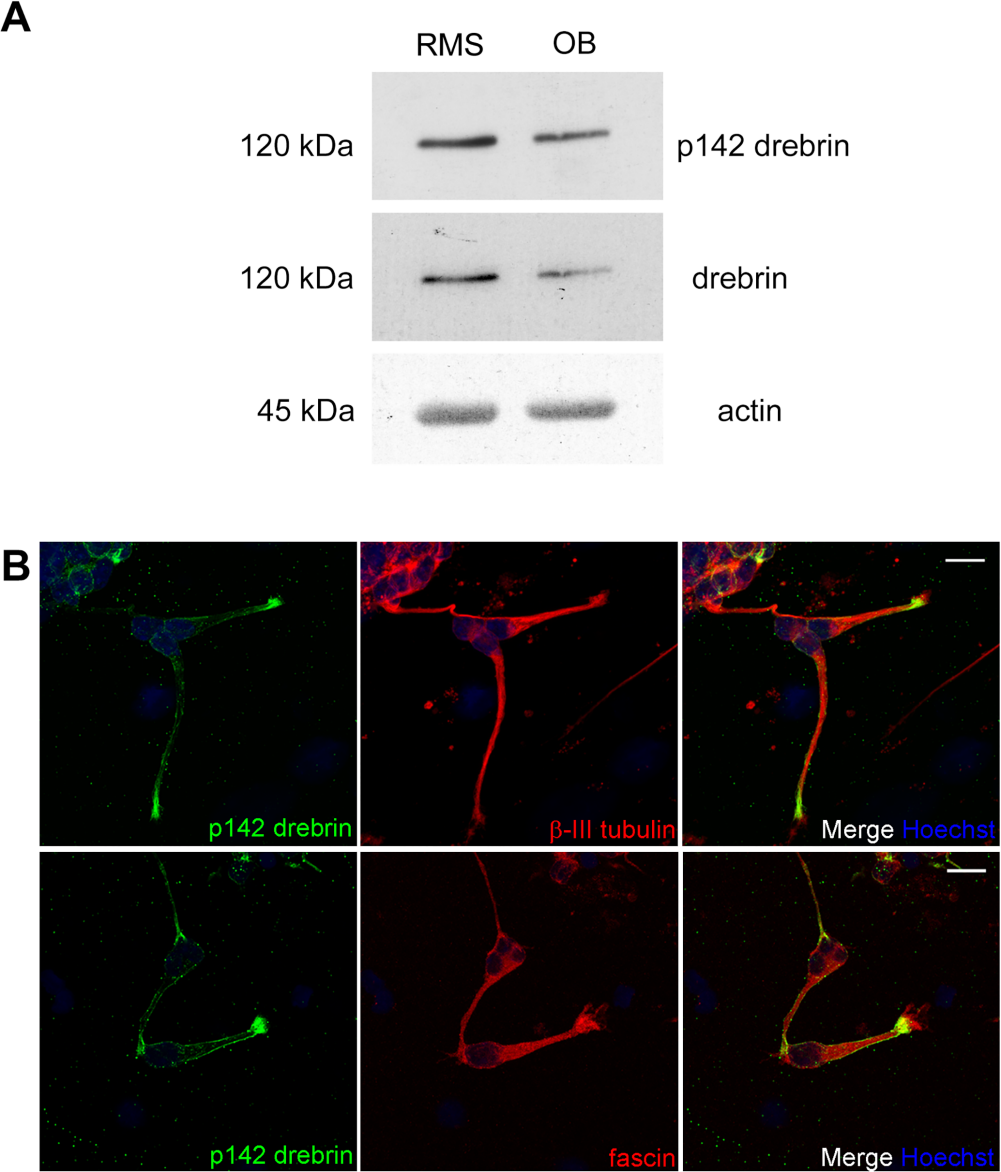
Localization of pS142-drebrin. (A) Drebrin phosphorylated on S142 can be detected in RMS and OB homogenates from P7 rat pups by Western blot using an anti-pS142-drebrin antibody. Actin is shown as a loading control. (B, top) Rat RMS neuroblasts were immunostained for pS142 drebrin (green) and βIII tubulin (red). pS142 drebrin is found along the membrane of the leading process and close to the tip of the leading process. (B, bottom) Neuroblasts were immunostained for drebrin pS142 (green) and fascin (red). Fascin and drebrin show colocalisation at the basal region of filopodia. Nuclei are stained with Hoechst (blue). Scale bars: 10 μm.

To study the localization of pS142-drebrin, we used an antibody specifically recognizing this phosphorylation site (S3 Fig) [15]. We detected strong pS142-drebrin signal on Western blots obtained from P7 rat RMS and OB homogenates (Fig 6A). To analyse the intracellular localization of pS142-drebrin, rat RMS explants were embedded in Matrigel and immunostained with the anti-pS142-drebrin antibody (Fig 6B). Phospho-S142 drebrin was detected along the plasma membrane and at the tip of the leading process in a restricted region close to microtubule tips, (Fig 6B, top row). pS142-drebrin also colocalised with fascin at the base of peripheral filopodia (Fig 6B, bottom row).

**Fig 7:**
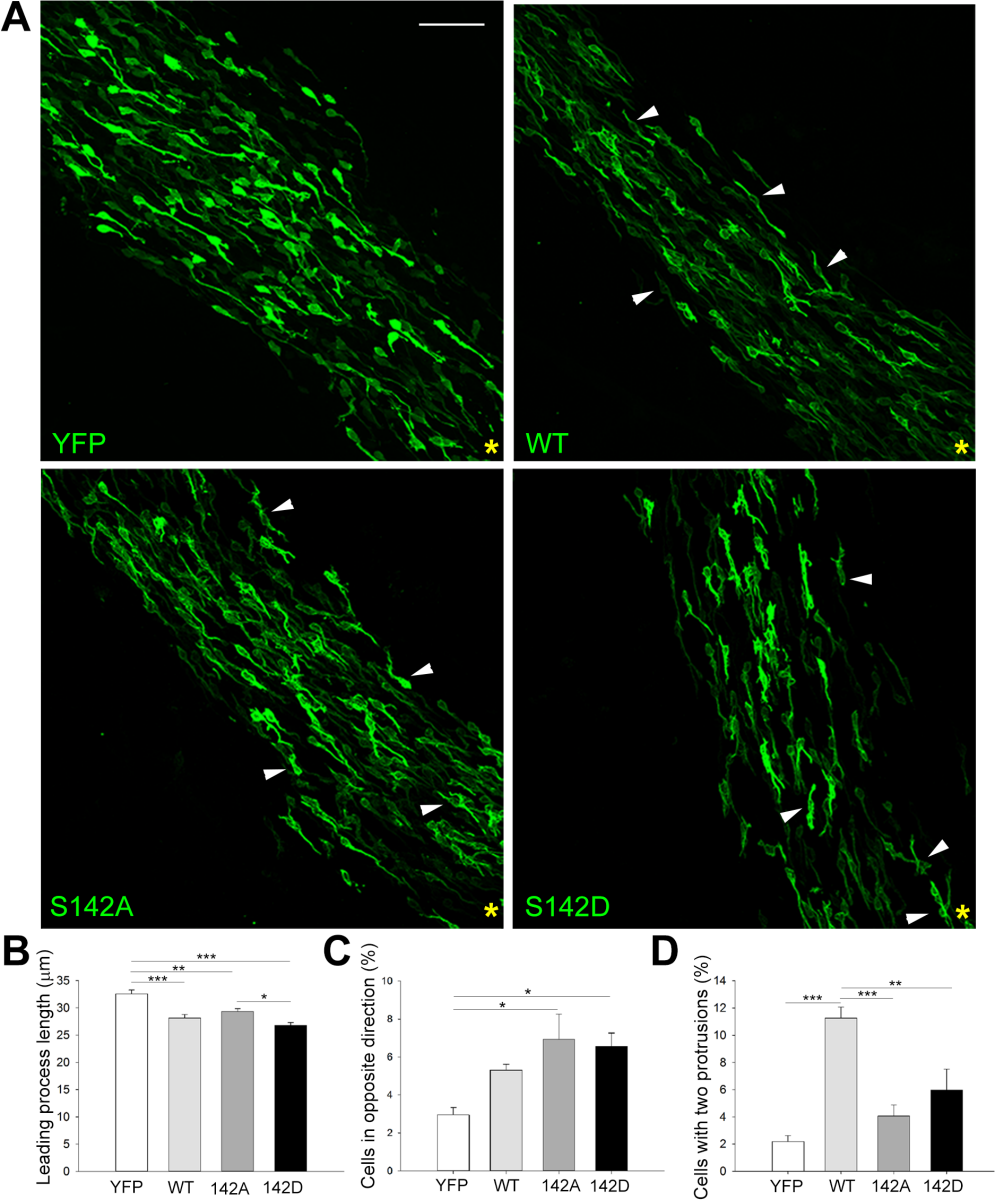
Drebrin phosphorylation on S142 regulates neuroblast morphology and orientation *in vivo*. (A) Sagittal brain slices were immunostained for YFP 5 days after *in vivo* electroporation of plasmids encoding YFP-tagged wt, S142A or S142D drebrin and imaged with a confocal microscope. The empty vector encoding only YFP served as control. The majority of control cells display a single leading process oriented towards the OB (yellow asterisk). Many neuroblasts overexpressing wt drebrin display a bipolar morphology with two diametrically opposite protrusions (one towards the OB and one towards the SVZ) (wt, arrowheads and D). Overexpression of S142A or S142D drebrin significantly increases the percentage of misoriented cells (pointing towards the SVZ instead of the OB, arrowheads) compared to control or wt drebrin (C). (B) Quantitative morphological analysis also shows a decrease in leading process length for wt, S142A and S142D compared to the control (mean ± SEM; n = 8 brains for empty vector; n = 5 brains for wt, S142A, and S142D drebrin; *P<0.05, **P<0.01, ***P<0.001). Scale bar: 50 μm.

To further explore the effect of drebrin overexpression and its phosphorylation on S142 on neuroblast morphology and directionality *in vivo*, brains from mice electroporated at P2 with plasmids encoding YFP-tagged wild type (wt) drebrin, non-phosphorylatable (S142A) or phosphomimetic (S142D) drebrin were fixed and immunostained for YFP 5 days post-electroporation. The empty vector encoding only YFP was used as a control. Neuroblasts expressing wt, S142A and S142D drebrin displayed a visibly different morphology compared to control cells (Fig 7A), showing shorter leading processes (Fig 7B). More than 95% of control cells have a protrusion oriented towards the OB. Importantly, overexpression of either S142A or S142D drebrin significantly affected neuroblast orientation, while expression of wt drebrin did not have a significant effect (Fig 7A, arrowheads in bottom panels, and Fig 7C). Intriguingly, overexpression of wt drebrin caused a substantial increase in the percentage of cells displaying two diametrically opposite protrusions extending from the cell body (one oriented towards the OB and the other towards the SVZ) (Fig 7A, arrowheads in upper right panel, and Fig 7D). Overall, these results support an important role for drebrin in regulating the unipolar morphology of neuroblasts and suggest that a tight regulation of a phospho/dephospho cycle on S142 of drebrin is required for proper neuroblast orientation during polarized migration along the RMS.

### Altering drebrin levels affects RMS neuroblast migration and newborn neuron distribution in the OB

**Fig 8:**
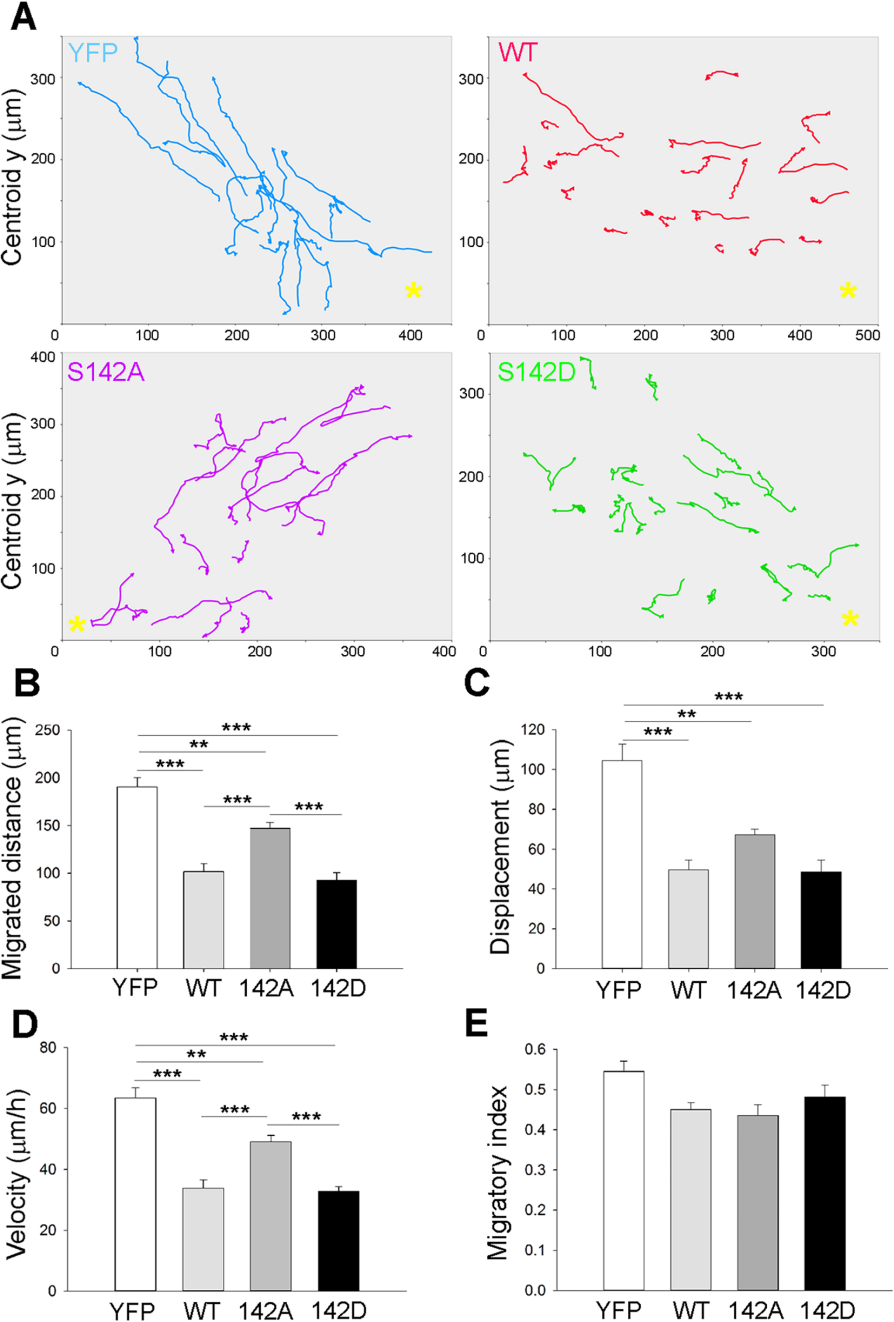
Altering levels of drebrin and pS142 phosphorylation affects neuroblast migration along the RMS. P2 mice were electroporated *in vivo* with plasmids encoding YFP, or YFP-tagged wt, S142A, or S142D drebrin. Fluorescently-labelled RMS neuroblasts were imaged 5 days later in acute brain slice cultures over a period of 3 hours. (A) Representative migration paths of neuroblasts expressing YFP empty vector, YFP-tagged wt, S142A, or S142D drebrin. Yellow asterisks mark the location of the OB. Quantitative tracking analysis reveals that neuroblasts overexpressing any one of the drebrin versions display a lower migratory distance (B), displacement (C), speed (D) and a trend towards a lower migratory index (E) compared to control cells expressing only YFP (mean ± SEM; n = 6 brains for control; n = 5 brains for wt; and n = 4 brains for S142A and n = 5 for S142D; **P<0.01, ***P<0.001).

To investigate the effect of altering drebrin and pS142-drebrin levels on neuroblast dynamics, we imaged RMS neuroblasts in brain slice cultures obtained from mouse pups electroporated with control empty YFP vector, or YFP-tagged wt, S142A or S142D drebrin 5 days after electroporation. Quantitative tracking analysis revealed that control YFP-expressing cells displayed a more directed motile behaviour compared to wt, S142A or S142D drebrin-expressing cells (Fig 8A). Neuroblasts overexpressing wt, S142A or S142D drebrin showed significantly decreased migration distance (Fig 8B), displacement (Fig 8C), and velocity (Fig 8D) compared to control cells and tended to have a lower migratory index (i.e. the ratio between net displacement and total distance covered [35]) (Fig 8E), meaning they tended to be more “exploratory” in their movement.

**Fig 9:**
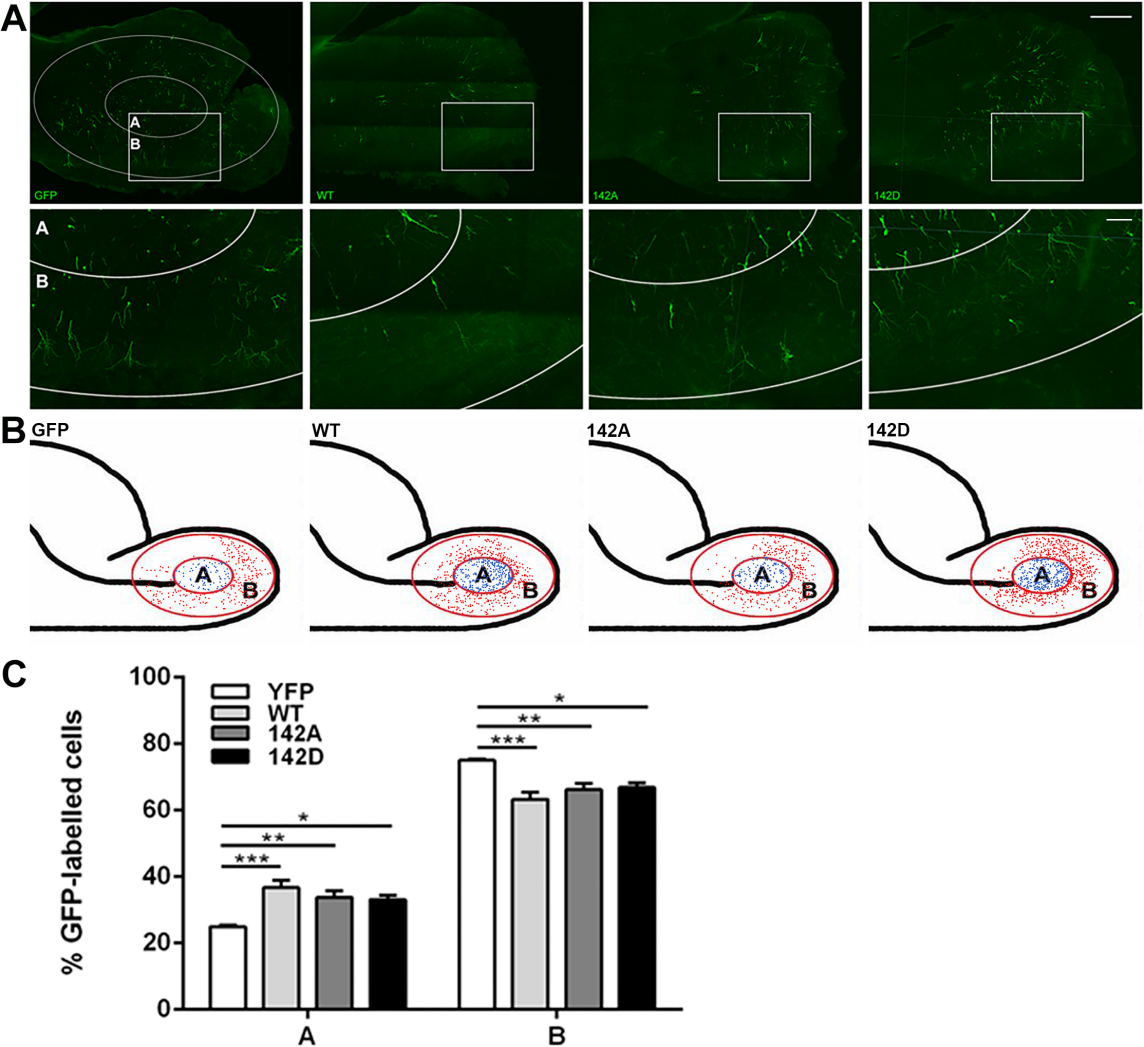
Altering levels of drebrin and pS142 phosphorylation of drebrin affects neuroblast distribution in the OB. P2 mice were electroporated in the lateral ventricle with plasmids encoding YFP or YFP-tagged wt, S142A or S142D drebrin. Sagittal brain slices were prepared 14 days later and immunostained for YFP. (A, top row) Representative confocal projections of sagittal brain slices showing overall labeling in the OB and overlaid with the mask used for analysis (area A, inner OB; area B, outer OB). High magnification pictures of the OB insets are shown in the bottom row. (B) Schematic diagrams of a typical quantification analysis using all OB sections from one brain for each condition. Cell bodies in areas A and B are represented as dots. (C) Overexpression of wt, S142A and S142D drebrin caused a significant cell accumulation in the inner OB compared to GFP (mean ± SEM; n = 3 brains for each construct; *P<0.05, **P<0.01, ***P<0.001). Scale bars: (A, top row), 500 μm; (A, bottom row), 100 μm.

To assess the long-term consequences of perturbing wt and pS142 drebrin levels, we analysed the distribution of YFP-positive cells found in the OB 14 days after *in vivo* electroporation (Fig 9). At this later time point, most of the control cells have radially migrated in the outer layer of the OB (area “B” in Fig 9A and 9B), and started to differentiate into interneurons. Almost 80% of control, YFP-labelled cells were found in the outer OB, while overexpression of wt, S142A or S142D drebrin caused a ~20% decrease in the percentage of cells found in the outer OB (Fig 9C). Therefore, altering drebrin levels and its phosphorylation levels on S142 ultimately disrupts the normal distribution of neuroblasts in the OB.

### Drebrin phosphorylation on S142 controls the stability of the leading process

**Fig 10:**
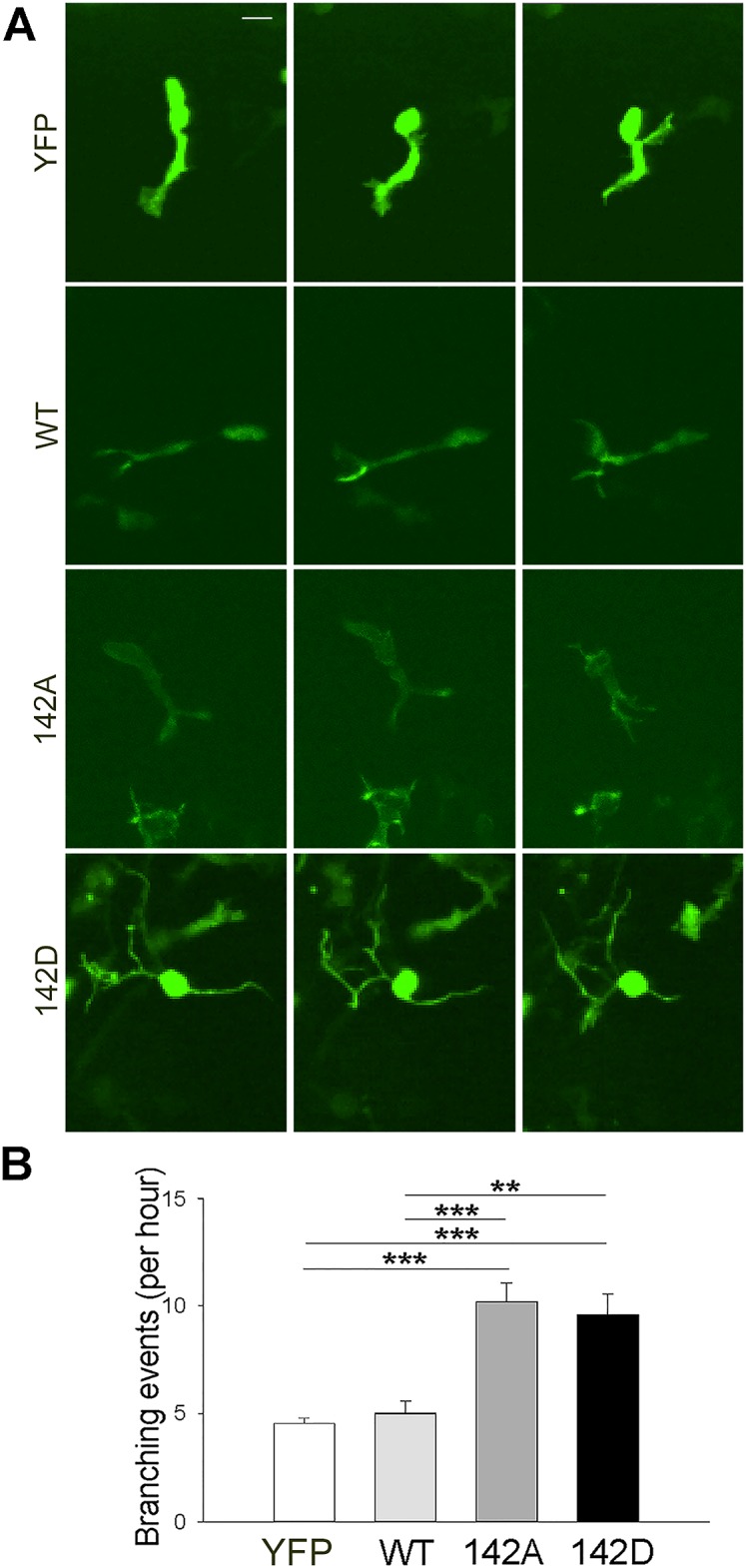
Phosphorylation of drebrin on Ser142 regulates the stability of the neuroblast leading process. P2 mice were electroporated in the lateral ventricle with plasmids encoding YFP-tagged wt, S142A or S142D drebrin. The empty vector encoding only YFP was used as control. Acute brain slices were prepared 5 d after electroporation. (A) Projections of spinning disk confocal z-stacks (corresponding to the same cell imaged at three different time points) showing representative migrating neuroblasts expressing YFP, YFP-tagged wt, 142A or 142D drebrin. (B) The branching frequency of migrating neuroblasts was visualized by confocal time-lapse imaging over one hour. RMS neuroblasts expressing 142A or 142D drebrin branched significantly more compared to cells expressing only YFP or wt drebrin (mean ± SEM; n = 6 brains for control; n = 5 brains for wt; n = 3 brains for 142A and 142D; **P<0.01, ***P<0.001). Scale bar: 10 μm.

To characterize the role of drebrin phosphorylation on S142, we monitored the branching frequency of neuroblasts in our time-lapse movies obtained from slice cultures of mouse pups electroporated with YFP, or YFP-tagged wt or phospho-drebrin mutants (Fig 10A). Interestingly, expression of either phospho-drebrin mutant doubled the frequency of branching events, while wt drebrin did not significantly affect branching compared to YFP-expressing control cells (Fig 10B). Taken together, these data show that drebrin expression levels must be regulated for efficient neuroblast migration, and suggest that a tightly regulated phosphorylation cycle of drebrin on S142 is required for the stability of the leading process to ensure the directed movement of neuroblasts along the RMS.

## Discussion

In this study we show that drebrin is necessary for the correct migration of SVZ-derived neuroblasts. Drebrin shRNA and siRNA-mediated knockdown disrupted neuroblast morphology and migration both *in vitro* and within the RMS. Overexpression of wt drebrin and its phospho-S142 mutants impaired tangential migration in the RMS and affected the distribution of neuroblasts in the OB. Furthermore, our results suggest that a dynamic, tightly regulated phospho-dephosphorylation cycle of drebrin on S142 maintains the stability of the leading process, contributing to efficient polarized migration along the RMS.

### Drebrin localisation in migratory neuroblasts

Drebrin is one of the major actin binding proteins in the brain, and only the E isoform is highly expressed in the adult rat RMS [19]. We extended these observations in the mouse model by confirming that drebrin is upregulated in the RMS also in early postnatal stages. According to out immunohistochemistry analysis, the great majority of drebrin-positive cells corresponds to DCX-expressing migratory neuroblasts, while little colocalisation was observed between drebrin and the astrocytic/stem cell marker GFAP or the proliferative progenitor marker Mash-1 (Fig 1). We recently found that another actin regulator, fascin, is also highly upregulated in DCX-positive neuroblasts and is required for their efficient migration along the RMS [23]. Interestingly, drebrin E may help suppress crosslinking of actin filaments by competing with the actin-bundling protein fascin [36], suggesting an important function for actin cytoskeletal remodeling in the transition from immobile to migratory neural progenitors in the mammalian SVZ.

In neuroblasts drebrin is found throughout the cell, but is especially concentrated in a region of the process tip overlapping with the basal region of fascin-enriched peripheral filopodia (Fig 2). This distribution is reminiscent of the one observed in post-mitotic developing neurons, where drebrin accumulates in the transition zone of growth cones [13, 18, 37], an area of active cytoskeletal remodeling. Here, drebrin can facilitate cross-linking between actin filaments and microtubules, stimulating neuritogenesis [15] and a similar function could promote effective actin/microtubule dynamics in migratory neuroblasts. A recent study has reported that drebrin is essential for the F-actin dependent entry of microtubules in dendritic spines of embryonic hippocampal neurons [38]. Whether drebrin is needed for entry of microtubules into the distal region of the leading process during neuroblast migration will require further investigation.

We found that pS142 drebrin is confined to the cell plasma membrane and the pre-terminal region of the leading process tip, co-localising with fascin at the proximal end of peripheral filopodia (Fig 6A). Previous studies described a more peripheral localisation of pS142 drebrin compared to unphosphorylated drebrin in the growth cones of embryonic cortical neurons, where microtubules terminate [15, 18]. It is tempting to speculate that while unphosphorylated drebrin acts as a destabilizer of actin bundles by inhibiting fascin binding to actin [36], pS142 drebrin may be able to bundle actin more efficiently by the “opening” of a second actin binding site, thus creating a stable base at the root of filopodia for fascin-mediated actin bundling. Moreover, pS142 drebrin, by exposing a domain that binds to EB3, could also facilitate the invasion of dynamic microtubules in the peripheral area of the tip of the leading process, allowing their growth along the existing filopodia [15].

The presence of pS142 drebrin on the plasma membrane suggests that drebrin phosphorylation could also regulate intercellular contacts in chains of migrating neuroblasts. Indeed, drebrin has been implicated in regulating adhesion in a number of contexts, including adherens junctions of epithelial cells, keratinocytes, basal cell carcinomas and endothelial cells [39–41]. In non-neuronal cells, drebrin can stabilise connexin-43-containing gap junctions [42]. Interestingly, gap junctions are also involved in neuronal migration and connexin-43 is required for efficient neuronal migration in the neocortex [43]. Connexin-43 is also highly expressed along the RMS (our unpublished data), suggesting an important role for gap junctions in RMS neuroblast motility. Cdk5 has been reported as the main kinase phosphorylating drebrin on S142 [15, 18]. Interestingly, since Cdk5 is required for the formation of neuroblast chains in the RMS [14], Cdk5-dependent phosphorylation of drebrin on S142 might be a key player in the dynamic coordination between cytoskeletal rearrangements and intercellular contacts required for efficient chain migration.

### The role of drebrin in neuroblast morphology

Drebrin can control morphology in different cell types, and its overexpression can increase the number of neurite-like processes and promote the formation of long protrusions [44–46]. Overexpression of drebrin in glioma cells alters cell morphology, leading to stellate cells with more projections [47]. In migrating oculomotor neurons drebrin depletion leads to a lack of leading process formation, while its overexpression promotes axon branching and alters growth cone shape [16]. In our system, RMS neuroblasts lacking drebrin are still able to extend a leading process, however they display substantial aberrant branching both *in vitro* and *in vivo*. The presence of drebrin may ensure proper cross-talk between the actin and microtubule cytoskeleton and intercellular adhesion in the RMS, thus contributing to maintain the typical unipolar morphology of migratory neuroblasts. Intriguingly, drebrin overexpression *in vivo* promoted the appearance of a bipolar shape with protrusions oriented in opposite directions, one towards the OB and one towards the SVZ, a very rare event in control conditions. Overall, these observations lead us to conclude that drebrin is not only required for the maintenance of a properly polarized leading process, but also that its expression must be highly regulated. The unpolarised morphology observed in drebrin-depleted neuroblasts may be linked to the alteration in the activation state of the small GTPase Cdc42, a master regulator of cell polarity and filopodia formation [48]. Interestingly, in synapses drebrin can interact with the Cdc42 scaffold protein Homer2 through its Homer binding domain, and the Drebrin-Homer-Cdc42 complex has been involved in the regulation of spine morphology and synaptic function [49, 50]. Monitoring Cdc42 activation state in neuroblasts overexpressing or lacking drebrin could help clarify a potential role of drebrin in the control of neuroblast polarity.

### The role of drebrin in neuroblast migration

Using *in vivo* postnatal electroporation and time-lapse imaging of cultured brain slices we have uncovered a novel, cell autonomous role for drebrin in SVZ-derived neuroblast migration. Drebrin regulates migration of oculomotor neurons [16] and invasiveness of glioma cells [17] by influencing cellular morphology. In oculomotor neurons absence of drebrin completely inhibits leading process formation and blocks their migration, while its overexpression causes aberrant migration. In SVZ-derived neuroblasts, both drebrin depletion and overexpression had similar effects on tangential migration in the RMS, leading to lower speed, shorter migrated distance and displacement, promoting a less directed movement. Drebrin could therefore contribute to highly regulated actin dynamics in RMS migratory neuroblasts, given its ability to influence the activity of other actin regulators such as the actin stabilizer tropomyosin, and to directly or indirectly control actin filament bundling [15, 16, 19, 36, 51]. Close inspection of actin dynamics in the leading process of neuroblasts with altered drebrin levels would provide further insights on this possibility.

Interestingly, overexpressing drebrin also affected SVZ-derived neuroblast distribution in the OB. Indeed, we observed a significant increase of cells in the OB core compared to control samples 14 days after electroporation, a time point when most of the electroporated neuroblasts should have reached the outer OB layers (Fig 7F and 7G). This suggests that drebrin may regulate the dispersion of chains at the rostral end of the RMS, when individual neuroblasts start to migrate radially in the OB, either by controlling cell-cell adhesion or by contributing to the response to environmental cues promoting radial migration, such as reelin or tenascin-R [8]. Alteration of drebrin levels could also impair the motility of radially migrating neuroblasts, and further investigations in appropriate inducible genetic deletion models (i.e. deleting drebrin once neuroblasts have reached the OB core) may provide insights into this interesting possibility. Given that neuroblast migration is essential for the proper maturation of neuroblasts into neurons in the OB [52], future studies will be required to clarify the contribution of drebrin to the subsequent integration of newborn neurons into the pre-existing synaptic network, especially considering the shift in drebrin localization towards putative synaptic contacts observed during the differentiation of neuroblasts into mature OB interneurons [19].

### The role of drebrin phosphorylation on S142 in neuroblast migration

A recent study highlighted the importance of a dynamic phosphorylation on two Cdk5-dependent phosphorylation sites identified on drebrin A (S142 and S342) in the radial migration of neurons during cortex development [18]. Cdk5 is a kinase crucial for many aspects of neuronal development, including neuronal morphogenesis, migration, axonal pathfinding and synaptic plasticity [53]. Importantly, Cdk5 is also required for efficient migration of RMS neuroblasts in a cell-autonomous manner and genetic deletion of Cdk5 alters directionality, lowers speed and disrupts intercellular adhesion in neuroblast chains [14]. We found that overexpression of both phosphomimetic and non-phosphorylatable drebrin mutants altered neuroblast orientation and impaired migration to a similar extent (Figs 6 and 7). Importantly, they also doubled the branching frequency in migratory neuroblasts compared to wt drebrin. A plausible explanation for the fact that both phosphomutants exhibit the same phenotype is that a dynamic phospho-dephosphorylation cycle on S142 is required to ensure stability of the leading process.

Interestingly, increased process branching is also observed when other important cytoskeletal regulators are perturbed in RMS neuroblasts, as in the case of deletion of the microtubule-binding protein DCX or overexpression of fascin phosphomutants, which ultimately result in decreased migration rate [23, 32]. Therefore, maintaining the stability of the leading process is crucial for effective migration, and our results identify drebrin as a key player in this process. This could require not only the precise coordination of F-actin/microtubule interaction (as shown in neuronal growth cones), but also regulation of adhesion and the ability to rapidly respond to extracellular cues.

In migrating cortical neurons, dynamic Cdk5-dependent phosphorylation of drebrin may promote the transition from the multipolar to a bipolar stage, which is required for initiation of glial-guided radial migration [18]. We observed an alteration in neuroblast orientation in the RMS when both S142 drebrin phosphomutants were overexpressed (Fig 6D). Regulated phosphorylation of drebrin on S142 may therefore contribute to guide neuroblasts along the RMS, providing a molecular tuning mechanism allowing the control of their orientation. Potential candidate signals activating Cdk5 in neuroblasts could be growth factors like Glial-Derived Neurotrophic Factor (GDNF) or guidance molecules like Semaphorins [8, 54], however the extracellular cues regulating drebrin phosphorylation and activity in migrating neuroblasts remain to be identified. Moreover, besides Cdk5, other still unidentified kinases could phosphorylate drebrin on S142 and potential additional sites to control its activity [18, 55, 56].

In summary, our data demonstrate that drebrin is required for proper postnatal neurogenesis by ensuring the efficient migration of SVZ-derived neuroblasts. Our data also suggest that a dynamic, tightly regulated phospho-dephosphorylation of drebrin on S142 could maintain the correct orientation and stability of the neuroblast leading process. In the future, it will be important to identify the signaling pathways regulating drebrin function for the potential coordination of intercellular adhesion and cytoskeletal remodeling during neuroblast migration.

## Supporting Information

S1 Fig Depletion of drebrin in RMS migrating neuroblasts using siRNA and shRNA. Representative blots from lysates of rat RMS neuroblasts nucleofected with control or drebrin siRNA oligos (A) or shRNA (B) and cultured for 48 or 72 hours were probed for drebrin and actin (loading control). (C) Densitometric quantitative analysis shows a significant reduction of drebrin levels at both time points after siRNA oligo nucleofection, although the most significant reduction was seen at 72 hours (mean ± SEM; n = 3 independent experiments; **P<0.01, ***P<0.001). (D) Densitometric quantitative analysis shows a significant reduction of drebrin levels of ~60% at 48 hours and ~80% at 72 hours after shRNA nucleofection (mean ± SEM; n = 3 independent experiments; ***P<0.001). (E) Confocal image showing effective drebrin knockdown in migrating neuroblasts expressing drebrin shRNA-GFP (green) (arrowheads). Non-transfected, GFP-negative neuroblasts retain high drebrin expression (red) (arrows). The asterisk shows a cell with low GFP expression (low drebrin knockdown). Scale bar: 20 μm.
(TIF)

S2 Fig Pharmacological inhibition of drebrin impairs neuroblast migration *in vitro* and *ex vivo*. (A) Rat RMS explants embedded in Matrigel were incubated with normal culture medium as control or medium with 1 μM BTP for 18 hours, fixed and stained with the nuclear dye Hoechst. Representative pictures of control and BTP-treated explants are presented in inverted contrast grayscale for better clarity. Scale bar: 50 μm. (B) Quantitative analysis shows a ~50% decrease of migration distance in BTP-treated cells (mean ± SEM; n = 3 independent experiments; 15–20 explants were counted for each condition; **P<0.01). (C-F) Brain slice cultures were prepared 5 days after *in vivo* electroporation of pCX-EGFP. Slices were incubated with or without BTP (1 or 2 μM) for 1 hour prior to imaging and imaged every 3 minutes for 3 hours. Drugs were present throughout the imaging period. Both BTP concentrations significantly decreased neuroblast migrated distance (C), displacement (D), and velocity (E). (F) Incubation with BTP (2 μM) also caused a reduction in the percentage of migratory cells (mean ± SEM; n = 5 brains for control, n = 3 for BTP 1 μM and n = 7 for BTP 2 μM; *P<0.05).
(TIF)

S3 Fig Western blot showing specificity of the antibody targeting drebrin phosphorylated on S142. A lysate obtained from COS-7 cells transfected with drebrin-YFP is positive for pS142-drebrin, whereas lysates from untransfected COS-7 cells or COS-7 cells transfected with the non-phosphorylatable S142A drebrin mutant are not. GAPDH levels indicate comparable sample loading.
(TIF)

## References

[1] Doetsch F, Alvarez-Buylla A. Network of tangential pathways for neuronal migration in adult mammalian brain. Proc Natl Acad Sci U S A. 1996;93(25):14895–900. 8962152 10.1073/pnas.93.25.14895 PMC26233

[2] Luskin MB. Restricted proliferation and migration of postnatally generated neurons derived from the forebrain subventricular zone. Neuron. 1993;11(1):173–89. . 8338665 10.1016/0896-6273(93)90281-u

[3] Lois C, Alvarez-Buylla A. Long-distance neuronal migration in the adult mammalian brain. Science. 1994;264(5162):1145–8. . 8178174 10.1126/science.8178174

[4] Lledo PM, Alonso M, Grubb MS. Adult neurogenesis and functional plasticity in neuronal circuits. Nat Rev Neurosci. 2006;7(3):179–93. 10.1038/nrn1867 . 16495940

[5] Ernst A, Alkass K, Bernard S, Salehpour M, Perl S, Tisdale J, et al Neurogenesis in the striatum of the adult human brain. Cell. 2014;156(5):1072–83. 10.1016/j.cell.2014.01.044 . 24561062

[6] Sanai N, Nguyen T, Ihrie RA, Mirzadeh Z, Tsai HH, Wong M, et al Corridors of migrating neurons in the human brain and their decline during infancy. Nature. 2011;478(7369):382–6. 10.1038/nature10487 21964341 PMC3197903

[7] Curtis MA, Eriksson PS, Faull RL. Progenitor cells and adult neurogenesis in neurodegenerative diseases and injuries of the basal ganglia. Clin Exp Pharmacol Physiol. 2007;34(5–6):528–32. 10.1111/j.1440-1681.2007.04609.x . 17439428

[8] Lalli G. Extracellular signals controlling neuroblast migration in the postnatal brain. Adv Exp Med Biol. 2014;800:149–80. 10.1007/978-94-007-7687-6_9 . 24243105

[9] Kojima N, Kato Y, Shirao T, Obata K. Nucleotide sequences of two embryonic drebrins, developmentally regulated brain proteins, and developmental change in their mRNAs. Brain Res. 1988;464(3):207–15. . 3208110 10.1016/0169-328x(88)90027-7

[10] Shirao T, Kojima N, Terada S, Obata K. Expression of three drebrin isoforms in the developing nervous system. Neurosci Res Suppl. 1990;13:S106–11. . 2259478 10.1016/0921-8696(90)90039-6

[11] Kojima N, Shirao T, Obata K. Molecular cloning of a developmentally regulated brain protein, chicken drebrin A and its expression by alternative splicing of the drebrin gene. Brain Res Mol Brain Res. 1993;19(1–2):101–14. . 8361332 10.1016/0169-328x(93)90154-h

[12] Shirao T, Kojima N, Kato Y, Obata K. Molecular cloning of a cDNA for the developmentally regulated brain protein, drebrin. Brain Res. 1988;464(1):71–4. . 3179746 10.1016/0169-328x(88)90020-4

[13] Geraldo S, Khanzada UK, Parsons M, Chilton JK, Gordon-Weeks PR. Targeting of the F-actin-binding protein drebrin by the microtubule plus-tip protein EB3 is required for neuritogenesis. Nat Cell Biol. 2008;10(10):1181–9. 10.1038/ncb1778 . 18806788

[14] Hirota Y, Ohshima T, Kaneko N, Ikeda M, Iwasato T, Kulkarni AB, et al Cyclin-dependent kinase 5 is required for control of neuroblast migration in the postnatal subventricular zone. J Neurosci. 2007;27(47):12829–38. 10.1523/JNEUROSCI.1014-07.2007 . 18032654 PMC6673299

[15] Worth DC, Daly CN, Geraldo S, Oozeer F, Gordon-Weeks PR. Drebrin contains a cryptic F-actin-bundling activity regulated by Cdk5 phosphorylation. J Cell Biol. 2013;202(5):793–806. 10.1083/jcb.201303005 23979715 PMC3760615

[16] Dun XP, Bandeira de Lima T, Allen J, Geraldo S, Gordon-Weeks P, Chilton JK. Drebrin controls neuronal migration through the formation and alignment of the leading process. Mol Cell Neurosci. 2012;49(3):341–50. 10.1016/j.mcn.2012.01.006 22306864 PMC3356577

[17] Terakawa Y, Agnihotri S, Golbourn B, Nadi M, Sabha N, Smith CA, et al The role of drebrin in glioma migration and invasion. Exp Cell Res. 2013;319(4):517–28. 10.1016/j.yexcr.2012.11.008 . 23201135

[18] Tanabe K, Yamazaki H, Inaguma Y, Asada A, Kimura T, Takahashi J, et al Phosphorylation of drebrin by cyclin-dependent kinase 5 and its role in neuronal migration. PLoS One. 2014;9(3):e92291 10.1371/journal.pone.0092291 24637538 PMC3956921

[19] Song M, Kojima N, Hanamura K, Sekino Y, Inoue HK, Mikuni M, et al Expression of drebrin E in migrating neuroblasts in adult rat brain: coincidence between drebrin E disappearance from cell body and cessation of migration. Neuroscience. 2008;152(3):670–82. 10.1016/j.neuroscience.2007.10.068 . 18304746

[20] Bron R, Eickholt BJ, Vermeren M, Fragale N, Cohen J. Functional knockdown of neuropilin-1 in the developing chick nervous system by siRNA hairpins phenocopies genetic ablation in the mouse. Dev Dyn. 2004;230(2):299–308. 10.1002/dvdy.20043 . 15162508

[21] Falenta K, Gajendra S, Sonego M, Doherty P, Lalli G. Nucleofection of Rodent Neuroblasts to Study Neuroblast Migration In vitro. J Vis Exp. 2013;(81). 10.3791/50989 . 24300093 PMC3990830

[22] Sonego M, Zhou Y, Oudin MJ, Doherty P, Lalli G. In vivo Postnatal Electroporation and Time-lapse Imaging of Neuroblast Migration in Mouse Acute Brain Slices. J Vis Exp. 2013;(81). 10.3791/50905 . 24326479 PMC3992024

[23] Sonego M, Gajendra S, Parsons M, Ma Y, Hobbs C, Zentar MP, et al Fascin regulates the migration of subventricular zone-derived neuroblasts in the postnatal brain. J Neurosci. 2013;33(30):12171–85. 10.1523/JNEUROSCI.0653-13.2013 23884926 PMC3721833

[24] Das A, Gajendra S, Falenta K, Oudin MJ, Peschard P, Feng S, et al RalA promotes a direct exocyst-Par6 interaction to regulate polarity in neuronal development. J Cell Sci. 2014;127(Pt 3):686–99. 10.1242/jcs.145037 24284074 PMC4007768

[25] Nam SC, Kim Y, Dryanovski D, Walker A, Goings G, Woolfrey K, et al Dynamic features of postnatal subventricular zone cell motility: a two-photon time-lapse study. J Comp Neurol. 2007;505(2):190–208. 10.1002/cne.21473 . 17853439

[26] Doetsch F. A niche for adult neural stem cells. Curr Opin Genet Dev. 2003;13(5):543–50. . 14550422 10.1016/j.gde.2003.08.012

[27] Scholzen T, Gerdes J. The Ki-67 protein: from the known and the unknown. J Cell Physiol. 2000;182(3):311–22. 10.1002/(SICI)1097-4652(200003)182:3<311::AID-JCP1>3.0.CO;2-9 . 10653597

[28] Kee N, Sivalingam S, Boonstra R, Wojtowicz JM. The utility of Ki-67 and BrdU as proliferative markers of adult neurogenesis. J Neurosci Methods. 2002;115(1):97–105. . 11897369 10.1016/s0165-0270(02)00007-9

[29] Oudin MJ, Gajendra S, Williams G, Hobbs C, Lalli G, Doherty P. Endocannabinoids regulate the migration of subventricular zone-derived neuroblasts in the postnatal brain. J Neurosci. 2011;31(11):4000–11. 10.1523/JNEUROSCI.5483-10.2011 . 21411643 PMC6623539

[30] Riedl J, Crevenna AH, Kessenbrock K, Yu JH, Neukirchen D, Bista M, et al Lifeact: a versatile marker to visualize F-actin. Nat Methods. 2008;5(7):605–7. 10.1038/nmeth.1220 18536722 PMC2814344

[31] Boutin C, Diestel S, Desoeuvre A, Tiveron MC, Cremer H. Efficient in vivo electroporation of the postnatal rodent forebrain. PLoS One. 2008;3(4):e1883 10.1371/journal.pone.0001883 18382666 PMC2270900

[32] Koizumi H, Higginbotham H, Poon T, Tanaka T, Brinkman BC, Gleeson JG. Doublecortin maintains bipolar shape and nuclear translocation during migration in the adult forebrain. Nat Neurosci. 2006;9(6):779–86. 10.1038/nn1704 . 16699506

[33] Mercer JC, Qi Q, Mottram LF, Law M, Bruce D, Iyer A, et al Chemico-genetic identification of drebrin as a regulator of calcium responses. Int J Biochem Cell Biol. 2010;42(2):337–45. 10.1016/j.biocel.2009.11.019 19948240 PMC2846297

[34] Zitt C, Strauss B, Schwarz EC, Spaeth N, Rast G, Hatzelmann A, et al Potent inhibition of Ca2+ release-activated Ca2+ channels and T-lymphocyte activation by the pyrazole derivative BTP2. J Biol Chem. 2004;279(13):12427–37. 10.1074/jbc.M309297200 . 14718545

[35] Comte I, Kim Y, Young CC, van der Harg JM, Hockberger P, Bolam PJ, et al Galectin-3 maintains cell motility from the subventricular zone to the olfactory bulb. J Cell Sci. 2011;124(Pt 14):2438–47. 10.1242/jcs.079954 21693585 PMC3124373

[36] Sasaki Y, Hayashi K, Shirao T, Ishikawa R, Kohama K. Inhibition by drebrin of the actin-bundling activity of brain fascin, a protein localized in filopodia of growth cones. J Neurochem. 1996;66(3):980–8. . 8769857 10.1046/j.1471-4159.1996.66030980.x

[37] Mizui T, Kojima N, Yamazaki H, Katayama M, Hanamura K, Shirao T. Drebrin E is involved in the regulation of axonal growth through actin-myosin interactions. J Neurochem. 2009;109(2):611–22. 10.1111/j.1471-4159.2009.05993.x . 19222710

[38] Merriam EB, Millette M, Lumbard DC, Saengsawang W, Fothergill T, Hu X, et al Synaptic regulation of microtubule dynamics in dendritic spines by calcium, F-actin, and drebrin. J Neurosci. 2013;33(42):16471–82. 10.1523/JNEUROSCI.0661-13.2013 24133252 PMC3797370

[39] Peitsch WK, Grund C, Kuhn C, Schnolzer M, Spring H, Schmelz M, et al Drebrin is a widespread actin-associating protein enriched at junctional plaques, defining a specific microfilament anchorage system in polar epithelial cells. Eur J Cell Biol. 1999;78(11):767–78. 10.1016/S0171-9335(99)80027-2 . 10604653

[40] Peitsch WK, Hofmann I, Bulkescher J, Hergt M, Spring H, Bleyl U, et al Drebrin, an actin-binding, cell-type characteristic protein: induction and localization in epithelial skin tumors and cultured keratinocytes. J Invest Dermatol. 2005;125(4):761–74. 10.1111/j.0022-202X.2005.23793.x . 16185277

[41] Rehm K, Panzer L, van Vliet V, Genot E, Linder S. Drebrin preserves endothelial integrity by stabilizing nectin at adherens junctions. J Cell Sci. 2013;126(Pt 16):3756–69. 10.1242/jcs.129437 . 23750010

[42] Butkevich E, Hulsmann S, Wenzel D, Shirao T, Duden R, Majoul I. Drebrin is a novel connexin-43 binding partner that links gap junctions to the submembrane cytoskeleton. Curr Biol. 2004;14(8):650–8. 10.1016/j.cub.2004.03.063 . 15084279

[43] Cina C, Maass K, Theis M, Willecke K, Bechberger JF, Naus CC. Involvement of the cytoplasmic C-terminal domain of connexin43 in neuronal migration. J Neurosci. 2009;29(7):2009–21. 10.1523/JNEUROSCI.5025-08.2009 . 19228955 PMC6666339

[44] Shirao T, Hayashi K, Ishikawa R, Isa K, Asada H, Ikeda K, et al Formation of thick, curving bundles of actin by drebrin A expressed in fibroblasts. Exp Cell Res. 1994;215(1):145–53. 10.1006/excr.1994.1326 . 7957662

[45] Hayashi K, Shirao T. Change in the shape of dendritic spines caused by overexpression of drebrin in cultured cortical neurons. J Neurosci. 1999;19(10):3918–25. . 10234022 10.1523/JNEUROSCI.19-10-03918.1999 PMC6782714

[46] Keon BH, Jedrzejewski PT, Paul DL, Goodenough DA. Isoform specific expression of the neuronal F-actin binding protein, drebrin, in specialized cells of stomach and kidney epithelia. J Cell Sci. 2000;113 Pt 2:325–36. . 10633083 10.1242/jcs.113.2.325

[47] Peitsch WK, Bulkescher J, Spring H, Hofmann I, Goerdt S, Franke WW. Dynamics of the actin-binding protein drebrin in motile cells and definition of a juxtanuclear drebrin-enriched zone. Exp Cell Res. 2006;312(13):2605–18. 10.1016/j.yexcr.2006.04.017 . 16780834

[48] Etienne-Manneville S, Hall A. Rho GTPases in cell biology. Nature. 2002;420(6916):629–35. 10.1038/nature01148 . 12478284

[49] Shiraishi-Yamaguchi Y, Sato Y, Sakai R, Mizutani A, Knopfel T, Mori N, et al Interaction of Cupidin/Homer2 with two actin cytoskeletal regulators, Cdc42 small GTPase and Drebrin, in dendritic spines. BMC Neurosci. 2009;10:25 10.1186/1471-2202-10-25 19309525 PMC2666743

[50] Dun XP, Chilton JK. Control of cell shape and plasticity during development and disease by the actin-binding protein Drebrin. Histol Histopathol. 2010;25(4):533–40. . 20183806 10.14670/HH-25.533

[51] Ishikawa R, Hayashi K, Shirao T, Xue Y, Takagi T, Sasaki Y, et al Drebrin, a development-associated brain protein from rat embryo, causes the dissociation of tropomyosin from actin filaments. J Biol Chem. 1994;269(47):29928–33. . 7961990

[52] Belvindrah R, Nissant A, Lledo PM. Abnormal neuronal migration changes the fate of developing neurons in the postnatal olfactory bulb. J Neurosci. 2011;31(20):7551–62. 10.1523/JNEUROSCI.6716-10.2011 . 21593340 PMC6622602

[53] Kawauchi T. Cdk5 regulates multiple cellular events in neural development, function and disease. Dev Growth Differ. 2014;56(5):335–48. 10.1111/dgd.12138 . 24844647

[54] Paratcha G, Ibanez CF, Ledda F. GDNF is a chemoattractant factor for neuronal precursor cells in the rostral migratory stream. Mol Cell Neurosci. 2006;31(3):505–14. 10.1016/j.mcn.2005.11.007 . 16380265

[55] Chew CS, Okamoto CT, Chen X, Thomas R. Drebrin E2 is differentially expressed and phosphorylated in parietal cells in the gastric mucosa. Am J Physiol Gastrointest Liver Physiol. 2005;289(2):G320–31. 10.1152/ajpgi.00002.2005 . 15790763

[56] Hayashi K, Ishikawa R, Kawai-Hirai R, Takagi T, Taketomi A, Shirao T. Domain analysis of the actin-binding and actin-remodeling activities of drebrin. Exp Cell Res. 1999;253(2):673–80. 10.1006/excr.1999.4663 . 10585290

