# Supplementary figures and images for: Drebrin Regulates Neuroblast Migration in the Postnatal Mammalian Brain
Source: PLoS One. 2015 May 6;10(5):e0126478. doi: 10.1371/journal.pone.0126478 (PMC4422745; doi:10.1371/journal.pone.0126478)

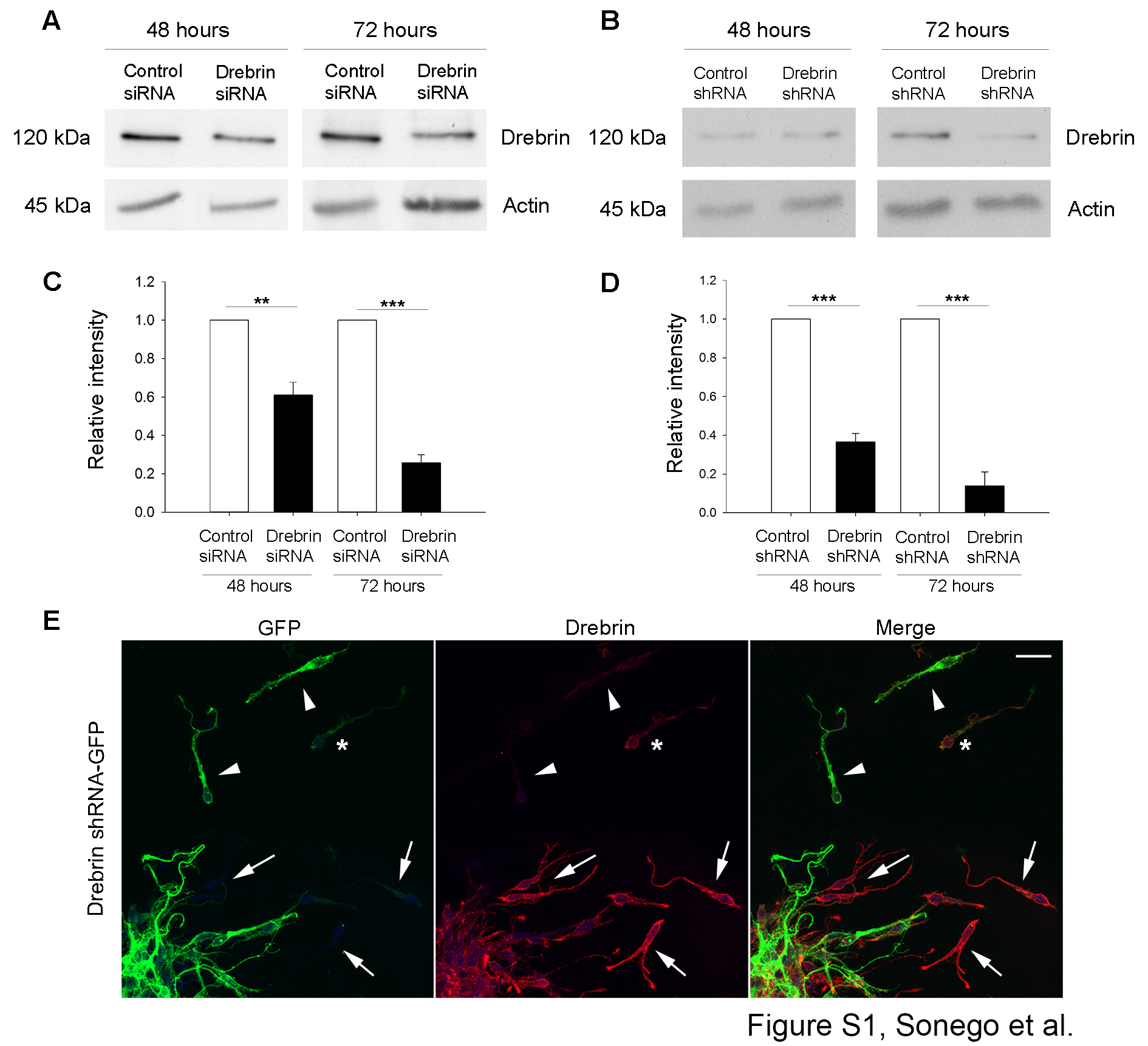

Supplement: S1 Fig — Representative blots from lysates of rat RMS neuroblasts nucleofected with control or drebrin siRNA oligos (A) or shRNA (B) and cultured for 48 or 72 hours were probed for drebrin and actin (loading control). (C) Densitometric quantitative analysis shows a significant reduction of drebrin levels at both time points after siRNA oligo nucleofection, although the most significant reduction was seen at 72 hours (mean ± SEM; n = 3 independent experiments; **P<0.01, ***P<0.001). (D) Densitometric quantitative analysis shows a significant reduction of drebrin levels of ~60% at 48 hours and ~80% at 72 hours after shRNA nucleofection (mean ± SEM; n = 3 independent experiments; ***P<0.001). (E) Confocal image showing effective drebrin knockdown in migrating neuroblasts expressing drebrin shRNA-GFP (green) (arrowheads). Non-transfected, GFP-negative neuroblasts retain high drebrin expression (red) (arrows). The asterisk shows a cell with low GFP expression (low drebrin knockdown). Scale bar: 20 μm. (TIF) [file pone.0126478.s001.tif]

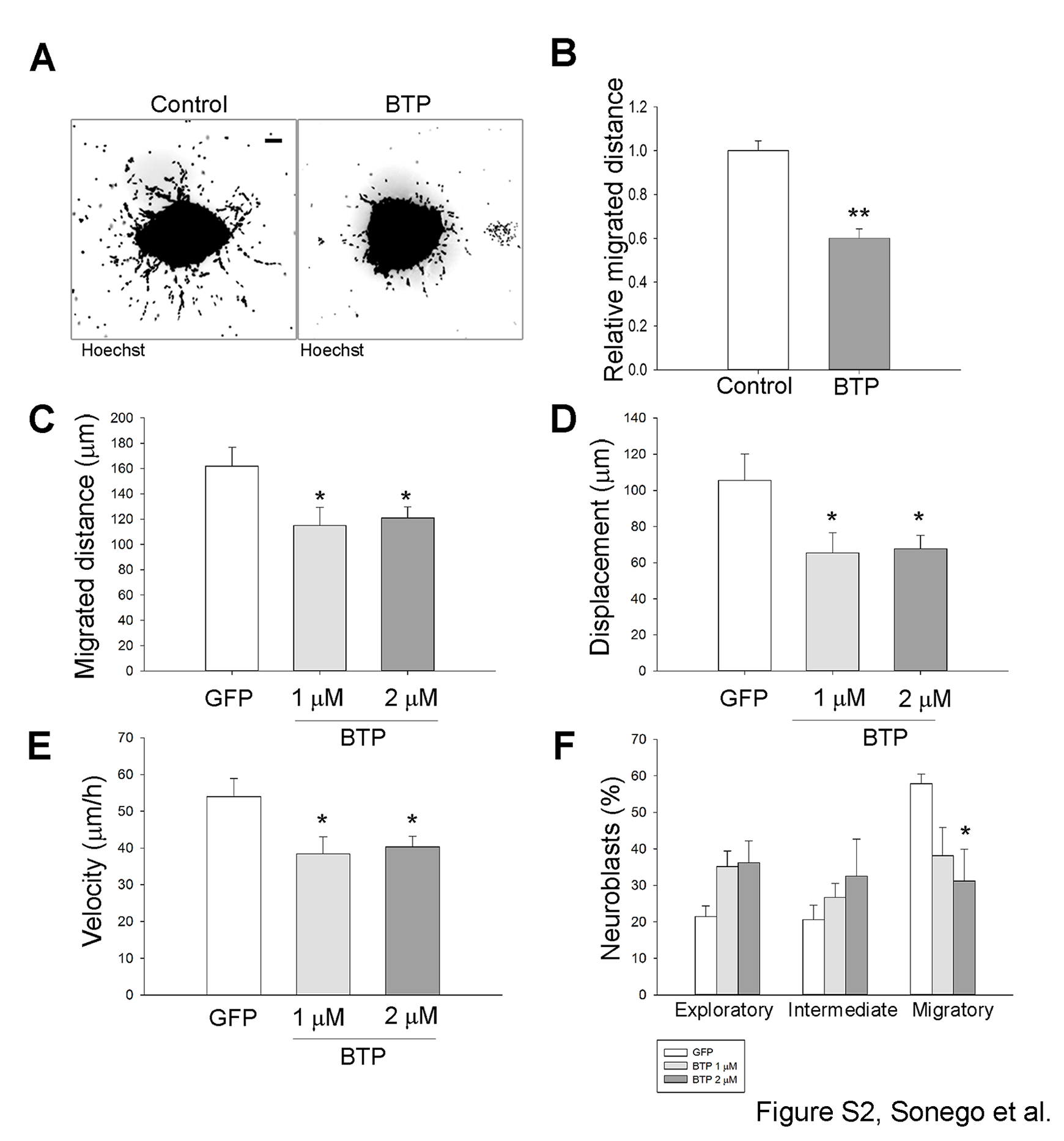

Supplement: S2 Fig — (A) Rat RMS explants embedded in Matrigel were incubated with normal culture medium as control or medium with 1 μM BTP for 18 hours, fixed and stained with the nuclear dye Hoechst. Representative pictures of control and BTP-treated explants are presented in inverted contrast grayscale for better clarity. Scale bar: 50 μm. (B) Quantitative analysis shows a ~50% decrease of migration distance in BTP-treated cells (mean ± SEM; n = 3 independent experiments; 15–20 explants were counted for each condition; **P<0.01). (C-F) Brain slice cultures were prepared 5 days after in vivo electroporation of pCX-EGFP. Slices were incubated with or without BTP (1 or 2 μM) for 1 hour prior to imaging and imaged every 3 minutes for 3 hours. Drugs were present throughout the imaging period. Both BTP concentrations significantly decreased neuroblast migrated distance (C), displacement (D), and velocity (E). (F) Incubation with BTP (2 μM) also caused a reduction in the percentage of migratory cells (mean ± SEM; n = 5 brains for control, n = 3 for BTP 1 μM and n = 7 for BTP 2 μM; *P<0.05). (TIF) [file pone.0126478.s002.tif]

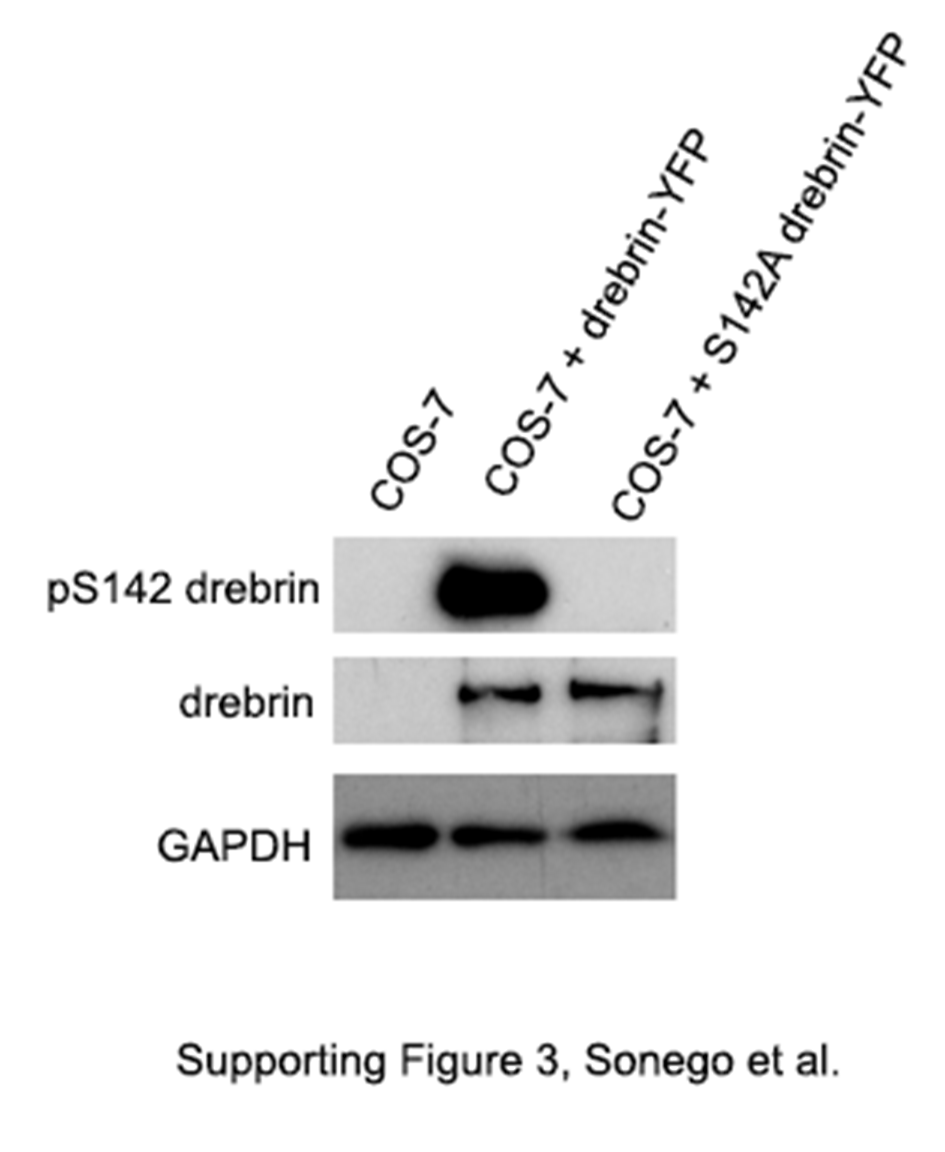

Supplement: S3 Fig — A lysate obtained from COS-7 cells transfected with drebrin-YFP is positive for pS142-drebrin, whereas lysates from untransfected COS-7 cells or COS-7 cells transfected with the non-phosphorylatable S142A drebrin mutant are not. GAPDH levels indicate comparable sample loading. (TIF) [file pone.0126478.s003.tif]
